# Two-Component Anomalous Hall and Nernst Effects in Anisotropic Fe_4**–** *x*_Ge_*x*_N Thin Films

**DOI:** 10.1021/acsomega.5c12420

**Published:** 2026-01-27

**Authors:** Robin K. Paul, Jakub Vít, Petr Levinský, Jiří Hejtmánek, Ondřej Kaman, Mariia Pashchenko, Lenka Kubíčková, Kyo-Hoon Ahn, Markéta Jarošová, Joris More Chevalier, Stanislav Cichoň, Tomáš Kmječ, Jaroslav Kohout, Marcus Hans, Stanislav Mráz, Jochen M. Schneider, Esmaeil Adabifiroozjaei, Leopoldo Molina-Luna, Oliver Gutfleisch, Imants Dirba, Karel Knížek

**Affiliations:** † Functional Materials, Institute of Materials Science, 529480 Technical University of Darmstadt, Peter-Grünberg-Str. 16, Darmstadt 64287, Germany; ‡ 86889 FZU − Institute of Physics of the CAS, Cukrovarnická 10, Praha 6 162 00, Czech Republic; § FZU − Institute of Physics of the CAS, Na Slovance 1999/2, Praha 8 182 00, Czech Republic; ∥ Faculty of Mathematics and Physics, 37740 Charles University, V Holešovičkách 747/2, Praha 8 180 00, Czech Republic; ⊥ Materials Chemistry, 9165 RWTH Aachen University, Kopernikusstr. 10, Aachen 52074, Germany; # Advanced Electron Microscopy Division, Institute of Materials Science, 26536 Technical University of Darmstadt, Peter-Grünberg-Str. 22, Darmstadt 64287, Germany

## Abstract

A series of thin films Fe_4–*x*_Ge_*x*_N (*x* = 0 – 1) were fabricated onto MgO substrates by magnetron sputtering with the aim of studying the possible enhancement of the anomalous Nernst effect (ANE), envisaged based on density functional theory (DFT) calculations. The Nernst and Hall effects of the series were systematically analyzed, complemented with resistivity, magnetic, electron microscopy, and Mössbauer experiments, and DFT calculations including elastic properties. The Fe_4_N phase crystallizes in the cubic symmetry with *Pm*3̅*m* space group, whereas a small tetragonal distortion is realized in Fe_4–*x*_Ge_*x*_N films for *x* > 0.35. From the comparison of the experimental isomer shift with DFT calculations, we conclude that Ge occupies the 4*b* site in the tetragonal *I*4/*mcm* structure. The ferromagnetic Curie temperature decreases rapidly from ∼750 K for *x* = 0 to ∼100 K for *x* = 1. The tetragonal samples with *x* = 0.8 and 1 display two-component behavior in the Hall and Nernst effects hysteresis loops, which can be analyzed as a sum of positive and negative loops with different saturation fields. This unusual behavior is a product of a combination of several factors: (1) coexistence of two different crystallographic orientations in the tetragonal thin film, namely with the majority of *c*-axis and minority of *a*-axis normal to the film surface; (2) opposite sign of the anomalous Hall and Nernst effects for the direction of magnetization along the *a*- and *c*-axes revealed by DFT calculation; and (3) the magnetocrystalline anisotropy characterized by an easy *ab*-plane, which is responsible for the different saturation fields for *a*- and *c*-axes. The maximum ANE was determined to be 0.9 μV/K for *x* = 0 at room temperature and −0.85 μV/K for *x* = 1 at *T* = 50 K. The rapid increase of ANE of Fe_3_GeN from low temperatures indicates that, were it not for its low Curie temperature, it could surpass the ANE of Fe_4_N. This observation is consistent with our theoretical assumptions and motivates further research of doped Fe_4_N for which ANE enhancement is predicted by DFT calculations.

## Graphical abstract

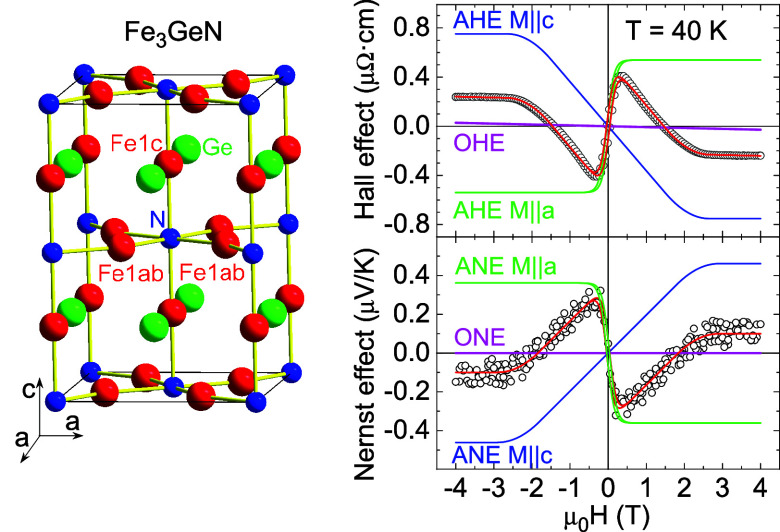

## Introduction

The application relevance of Fe_*x*_N (*x* ≥ 3) results from its favorable magnetic, electrical, and mechanical properties. Since it consists only of iron and nitrogen, both of which are cost-effective, abundant, and nontoxic elements, it provides cheap, environmentally friendly, and recyclable functional materials.^1–3^ The first applications of iron nitrides were related to their mechanical and chemical resistance and consisted of creating a coating layer that prevents metal corrosion and increases the mechanical strength of the steel. Further applications arose in connection with their excellent magnetic properties with Curie temperatures (*T*_*C*_) high above room temperature, which expanded their use as, e.g., high-density magnetic recording heads, magnetic storage media, strong permanent magnets, and, thanks to their low cytotoxicity, also as magnetic materials for biomedicine.^4–9^ The phase diagram of iron–nitrogen provides a variety of interesting materials with their magnetic properties tunable over a broad range depending on the nitrogen content. Magnetism correlates with the iron amount, starting from nonmagnetic FeN to ferromagnetic (FM) γ′-Fe_4_N^6^ with high magnetization and even further to α′-Fe_8_N.^3,10^ Perhaps the most attention is attracted by the ordered tetragonal superstructure α″-Fe_16_N_2_ ^11^ due to its unique combination of high saturation magnetization with enhanced magnetocrystalline anisotropy,^12^ which has been studied for multiple potential applications, such as rare-earth-free permanent magnets,^13^ two-phase nanocomposite magnets,^14^ as well as biomedical applications.^8^

The favorable magnetic properties, also inspired research on transverse magneto-transport properties, such as Hall and Nernst effects. Significant transverse thermoelectric properties were discovered in epitaxial Fe_4_N films on various substrates, and the coefficient of the anomalous Nernst effect (ANE) up to 2.8 μV/K was obtained.^15,16^ The anomalous Hall effect (AHE) has also been examined mainly on Fe_4_N samples in the form of thin layers; in particular, the dependence of AHE on the film thickness^17^ on the nitrogen content^18^ or the influence of Mn doping^19^ were investigated. Important insight was also gained from the systematic evaluation of anomalous Hall and Nernst conductivities (AHC and ANC) of the series of substituted transition metal nitrides and carbides, including substituted Fe_4_N.^20^ This discovery further expanded the possible application of iron nitrides for thermoelectric energy harvesting or heat-flux sensors.^21–23^ Concerning the application of thermoelectric conversion for electric power generation, the best materials with high ANE values currently available cannot yet compete with established thermoelectric modules using the Seebeck effect. However, in the case of heat flux sensors, ANE-based modules may be more suitable than Seebeck-based modules. Thanks to the transverse geometry of the Nernst effect, the signal is generated independently of the thickness of the material. As a result, the advantage of ANE-based modules resides in their smaller thickness and thus lower thermal resistance, while the sensitivity of both types of modules can reach comparable values.^22^

In our previous work, using ab initio density functional theory (DFT) combined with Berry curvature calculations, promising systems for ANE applications with an absolute value of ANC up to ∼8 A K^–1^ m^–1^ in substituted Fe_4_N have been identified.^24^ However, a solid solution in the required range is not available for most of the proposed substitutions. For our study, we have chosen the Fe_4–*x*_Ge_*x*_N series as it is one of the few series for which the existence of solid solutions up to *x* = 1 has been proven for bulk samples.

The first report on Ge substitution in Fe_4_N can be found in refs 25,26 . Tetragonal structure with space group *I*4/*mcm* was identified for Fe_3_GeN, which was also confirmed in subsequent works. Magnetic and structural properties of Fe_3_GeN_*y*_ (*y* ≤ 0.82) were later studied in ref 27, where the authors observed a significant decrease of magnetization and critical temperature with *y*. The symmetry changes from cubic *Pm*3̅*m* to tetragonal *I*4/*mcm* above *N* content *y* ∼ 0.3. Electrical transport including Hall effect of Fe_3_GeN was studied in ref 28. The authors reported the temperature evolution of the ordinary Hall effect, which was negative at room temperature, changed to positive above critical temperature *T_C_* ∼ 80 K and reverted to a negative value below *T_C_*. A positive anomalous Hall effect (AHE) is observed below *T_C_*. The detailed investigation of the magnetic properties of Fe_3_GeN revealed the ground magnetic state as frustrated ferromagnetic, resulting from a competition of local magnetic moments of iron 3d electronic state and itinerant covalent interactions of Fe–N bonds.^29,30^ This explanation is in accordance with theoretical (DFT) investigation, which determined that Fe in the apical site accounts for most of the magnetization and has a relatively stable moment, whereas the Fe in the equatorial site has a much smaller and much more flexible moment. Magnetism may then be described as local Fe-apical moments embedded in an itinerant Fe-equatorial background.^30,31^ The development of structural and magnetic properties was studied for the complete Fe_4–*x*_Ge_*x*_N_*y*_ series in ref 32, but the determined properties were affected by a significant decrease in N content in Ge-rich samples. In a similar study of the Fe_4–*x*_Ge_*x*_N series,^33^ the N content was not determined, but the reduction of the lattice parameter *c* and of the tetragonal deformation with Ge content suggest that the decrease in N content is comparable to the previous report.

This work aimed to prepare the series Fe_4–*x*_Ge_*x*_N up to *x* = 1 in the form of thin films, determine their transverse magneto-transport properties, namely, Nernst and Hall effects, and correlate them with theoretical predictions. The study is complemented by a systematic experimental investigation of microstructural, magnetic, and transport properties. Our work is the first in which this series has been prepared in the form of thin films, and the Nernst effect has been determined.

## Methods

Thin films were deposited using magnetron sputtering in a custom-built setup. Fe and Ge targets were cosputtered in a nitrogen-containing atmosphere onto MgO(001) substrates maintained at 400 °C. Ar and N_2_ were used as sputtering gases, with partial pressures of 4.2 × 10^–3^ and 0.8 × 10^–3^ mbar, respectively. The chamber base pressure was around 10^–7^ mbar, and the deposition pressure was set to 4.5 × 10^–3^ mbar. The target-to-substrate distance was fixed at 10 cm for all of the depositions. To obtain different Fe/Ge compositions, the sputtering power for the Fe target was held constant at 100 W, while the Ge target power was varied between 13 W and 25 W. After the deposition, a protective coating of Al was deposited on selected films using an Al target inside the chamber.

The phase purity and the degree of preferred orientation (epitaxial growth) of the thin films were checked by X-ray diffraction acquired on a powder Bruker D8 Advance diffractometer (CuKα radiation, Lynxeye XE-T position-sensitive detector). Lattice parameters were refined using the FullProf package.^34^ The surface of the thin films and their stoichiometry were checked by scanning electron microscopy (SEM) and energy-dispersive X-ray spectroscopy (EDX) using a Jeol JXA-8230 apparatus. Thin lamellae of the thin films for transmission electron microscopy (TEM) were prepared by focused ion beam (FIB) irradiation within an FEI Helios Nanolab 660 dual-beam microscope. The interpretation of the selected-area electron diffraction images was done by the use of the CrysTBox.^35^ The chemical composition of the inner region of the films was investigated by using atom probe tomography (APT).

The low-temperature (2–300 K) measurements of resistivity and Hall effect were performed using the four-probe method of the Electrical Transport Option (ETO) of the Physical Property Measurement System (PPMS, Quantum Design). The magnetic response of the samples within the temperature range of 5–380 K was measured using a superconducting quantum interference device (SQUID) magnetometer (MPMS-XL, Quantum Design). High-temperature (300–850 K) magnetic response was measured using the vibrating sample magnetometer (VSM) EZ9 (Microsense) in N_2_ (<570 K) or Ar (>570 K) atmosphere. The measurements were carried out in two macroscopically distinct orientations of the external magnetic field, i.e., perpendicular and parallel to the sample surface. The Nernst effect was measured using the Thermal Transport Option (TTO) option of PPMS and using a homemade apparatus employing the standard geometry in which the directions of magnetic field *B_z_*, thermal gradient ∇*T_x_*, and the resulting electric field *E_y_* are mutually perpendicular. The details of the measurement geometry are discussed in the Results and Discussion section. The longitudinal and transverse transport coefficients, namely, the longitudinal electrical conductivity σ_xx_, Seebeck coefficient *S_xx_*, Hall conductivity σ*_x__y_*, and Nernst coefficient *S_xy_* are linked with the Nernst conductivity α_xy_ by the formula

1 $$\alpha_{xy}=\sigma_{xx}S_{xy}+\sigma_{xy}S_{xx}$$

The ordinary Hall conductivity was calculated using BoltzTraP2.^36^ The details about the calculation of the anomalous Hall conductivity $\sigma_{xy}^{A}$ and the anomalous Nernst conductivity $\alpha_{xy}^{A}$ utilizing the concept of the Berry curvature can be found in ref 24. DFT calculations using the Vienna *Ab initio* Simulation Package (VASP)^37^ were carried out with a *k* mesh of 20 × 20 × 20 points and a plane-wave cutoff of 600 eV. The projector-augmented wave^38^ potentials with the generalized gradient approximation (GGA)^39^ were used. For comparison with Mössbauer spectroscopy, we have calculated a set of hyperfine parameters for *x* = 1 according to procedures applied in ref 40.

The mechanical properties were characterized by nanoindentation using a Hysitron TI-900 TriboIndenter. Room-temperature ^57^Fe conversion-electron Mössbauer spectra of the Fe_4–*x*_Ge_*x*_N samples with *x* = 0.5 and 1 were obtained in constant-acceleration mode using a ^57^Co/Rh source; the velocity scale was calibrated against a room-temperature spectrum of an α-Fe foil, and isomer shifts are reported relative to its centroid. More experimental details can be found in the Supporting Information.

## Results and Discussion

### Phase Formation

The formation of Fe_4–*x*_Ge_*x*_N phases (*x* = 0–1) in thin films on the MgO substrate has been proved by X-ray diffraction (XRD). All the measured thin films exhibit epitaxial growth, i.e., high degree of preferred orientation both perpendicular to the film surface and within the film plane. The structure of Fe_4_N (*x* = 0) could be inferred from the *fcc* close-packing of γ-Fe by inserting a nitrogen atom into the center of the cube, thus lowering the symmetry to *Pm*3̅*m*. Another possible description of the structure can be based on the antiperovskite-type ABX_3_, which could be recalled by rewriting the formula as FeNFe_3_. Within this description, the nitrogen is located in the position of a small cation B in the center of the BX_6_ (NFe_6_) octahedron, and the iron is in two crystallographic sites, namely, in the position of a big cation A (Fe2) and as ligands X (Fe1) of the BX_6_ (NFe_6_) octahedron, see the structure in Figure 1a. The epitaxial growth perpendicular to the film surface is confirmed by standard θ–2θ scan, where only *h*00 (or 00*l*) reflections are detected, see Figure S1a in the Supporting Information. The epitaxial growth within the film plane is confirmed by the azimuthal scan of 113 reflections; see Figure S1b in the Supporting Information, where the zero angle is aligned along the 100 direction of the MgO substrate. It shows 4 separate peaks confirming 4-fold symmetry within the plane. The azimuthal angle of 113 reflection is inclined by 45°, meaning that 100 directions of the thin film and the substrate are parallel.

**Figure 1:**
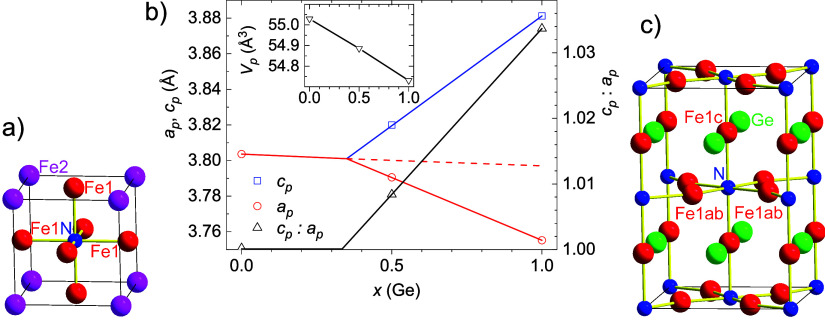
(a) Crystal structure of Fe_4_N. (b) Dependence of the lattice parameters of the primitive cell *a_p_* and *c_p_*, ratio of lattice parameters *c_p_*:*a_p_*, and the volume of the primitive cell *V_p_* on Ge content. (c) Crystal structure of Fe_3_GeN. Displayed atoms are Fe (red/magenta), Ge (green), and N (blue).

The symmetry of phases with higher *x* decreased to tetragonal *I*4/*mcm* structure having a 4 times bigger cell with *c*_*p*_:*a*_*p*_ > 1 (*a*_*p*_ and *c*_*p*_ are lattice parameters recalculated to a primitive cell with *Z* = 1), see Figure 1c. By comparing the experimental isomer shift (IS) of Mössbauer spectral components with our DFT calculations (see the Mössbauer spectroscopy section in the Supporting Information), we can conclude that Ge preferentially occupies the large cation Fe2 site (4*b* in Wyckoff notation). This finding is consistent with results reported for a polycrystalline sample.^32,33^ According to DFT calculations, the electron density at the Ge site is higher than that of a free atom; i.e., Ge acquires a negative oxidation state. It is in accordance with Ge preference for the large perovskite site, since the Ge anion should have a larger radius than the Fe cation. On the other hand, replacing the Fe–Fe metal bond with a stronger Ge–Fe ionic bond reduces the bond length and shrinks the cell volume, as observed by XRD. The site of Fe1 bonded to N is split into axial Fe1c and in-plane Fe1ab, see the structure in Figure 1c. From the extrapolation of the *c*_*p*_:*a*_*p*_ ratio, we estimate the border between cubic and tetragonal phase to *x* ∼ 0.35, see Figure 1b.

To compare the development of crystal symmetry and lattice parameters, it is important to consider both the Ge and N content. Both parent compounds Fe_3_Ge^27^ and Fe_4_N^32,33^ possess cubic *Pm*3̅*m* symmetry, and with increasing N content or with substitution of Ge for Fe, the symmetry changes to tetragonal *I*4/*mcm*, and the tetragonal distortion (*c*_*p*_:*a*_*p*_) increases both with N and Ge content. Varying nitrogen content is therefore the main reason for various limits of Ge­(*x*) stoichiometry for the transition to tetragonal symmetry, namely, *x* > 0.75 (nitrogen content *y* ∼ 0.65–0.34) in ref 32, *x* = 0.4–0.5 (*y* not determined) in ref 33, and *x* ∼ 0.35 (*y* ∼ 0.95–0.86, see Table 2) in our work. The higher nitrogen content in our films compared to ref 32 is consistent with our lower *x* of the onset of tetragonal deformation and the higher *c*_*p*_:*a*_*p*_ for *x* = 1 (1.034 vs 1.023). However, the additional influence of the strain induced by mismatch between the MgO substrate and the Fe_4–*x*_Ge_*x*_N film on the lattice parameters cannot be excluded.

The diffraction pattern for tetragonal *x* = 0.5 and 1 shows both 00*l* and *h*00 peaks (Figure S1a in the Supporting Information), meaning that epitaxial growth occurs with both *a*- and *c*-axes normal to the film surface. For *x* = 0.5, the ratio of *c*:*a* orientation is approximately 40%:60%, for *x* = 1 it is 80%:20%. This observation could be explained by a competition of two factors: 1) tetragonal phases typically grow with *c*-axis normal to the surface because of the growth kinetics and minimization of the surface energy. This tendency is enhanced for a bigger difference between *c* and *a*-axis. 2) lattice mismatch between Fe_4–*x*_Ge_*x*_N phases and MgO substrate, which has a NaCl-type structure with space group *Fm*3̅*m* and lattice parameter *a* = 4.21 Å. If we consider that Fe­(Ge) is bonded to oxygen in MgO at the interface and compare the distance between oxygens in MgO *d*_*O–O*_ = 2.98 Å, and the distance between Fe–Ge in Fe_3_GeN within *ab* plane *d*_*ab*_ ∼ 2.65 Å, and within *ac* plane *d*_*ac*_ ∼ 2.70 Å, there is 11% or 9% mismatch inducing strain on the nitride film. Thus, the orientation with the *c*-axis parallel with the film surface could at least partially decrease this strain. For *x* = 0.5, which has a smaller *c*:*a* ratio, the influence of both these factors is comparable. As the ratio *c*:*a* increases for *x* = 1, the influence of the first factor becomes more dominant and the percentage of *c*-axis normal to the surface increases. Structural data for the Fe_4–*x*_Ge_*x*_N films (*x* = 0, 0.5, and 1) are summarized in Table 1.

**Table 1:** Lattice Parameters and Preferred Orientation of the Fe_4‑x_Ge*_x_* N Thin Films

						Orientation	
*x*(Ge)	Space group	*a* (Å), *a*_*p*_ (Å)^a^	*c* (Å), *c*_*p*_ (Å)^a^	*V_p_* (Å^3^)^a^	*c_p_*:a*_p_* ^a^	00*l*	*h*00
0	*Pm*3̅*m*	3.804(1)		55.03(1)	1.000	100%	
0.5	*I*4/*mcm*	5.361(1)	7.640(2)	54.89(1)^a^	1.008^a^	40%	60%
		3.791(1)^a^	3.820(1)^a^				
1	*I*4/*mcm*	5.311(3)	7.762(2)	54.73(2)^a^	1.034^a^	80%	20%
		3.755(2)^a^	3.881(1)^a^				

a Recalculated to a primitive cell with *Z* = 1.

The rocking-curve scans of 200/002 reflection in Figure S1c show the degree of preferred orientation of thin films. The preferred orientation is higher for *x* = 0 and 1 (narrower peaks) than for *x* = 0.5. For *x* = 0.5, the large cation site is occupied randomly by Fe and Ge, which introduces a certain degree of disorder into the structure, while for *x* = 0 and 1, the occupancy of each site is close to 100% by individual ions.

### Microstructure

The stoichiometry of the films determined by averaging several points in SEM images using EDX is displayed in Table 2. The contents of Fe and Ge are close to the expected composition, while the nitrogen content is slightly lower, and the difference between nominal and determined content increases with *x*. The surface of the film visualized by backscattered electrons using SEM reveals an interesting pattern for *x* = 1, which consists of perpendicular, evenly spaced dark stripes that form an almost regular grid, see Figure 2. The stripes differ by a lower Ge content of *x* ∼ 0.85(4) from the rest of the sample with *x* ∼ 0.97(2). Let us note that the focus spot of the EDX is larger than the stripe width; therefore, the stripe composition is calculated by comparing the composition of the light area itself and of the mixture of the stripe and the light area. We estimate that the stripes comprise about 15–20% of the thin film volume, which would give an overall Ge content *x* ∼ 0.95. Since the volume of the stripes corresponds to the fraction of cells with the *a*-axis perpendicular to the film surface, we assign these stripes to this distinct preferred orientation. It would also explain the tendency toward lower Ge content in stripes, since lower *x* for cells oriented with their *a*-axis perpendicular to the film surface would decrease the difference between lattice parameters *a* and *c* and reduce the possible strain within the film plane, see lattice parameters in Figure 1. No such stripes were observed for *x* = 0 and 0.5, since there is no or small difference between *a* and *c* lattice parameters.

**Table 2:** Average Composition Determined by EDX

Nominal Ge (*x*)	Fe	Ge	N
0	4	0	0.97(2)
0.5	3.51(2)	0.49(2)	0.95(3)
1	3.05(2)	0.95(2)	0.86(3)

**Figure 2:**
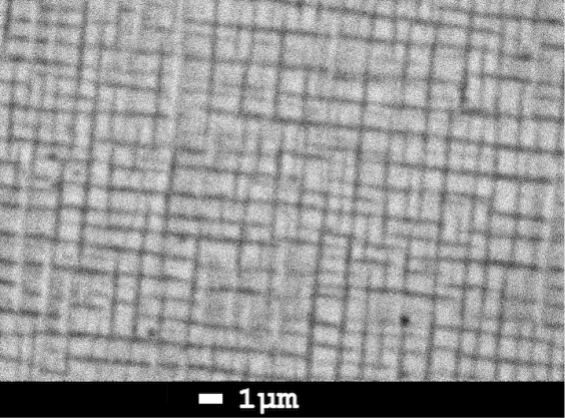
Backscattered electron image of the *x* = 1 film. The Ge content in dark stripes was determined as *x* ∼ 0.85, in the light area as *x* ∼ 0.97.

### Nanostructure

For deeper insight into the microstructure and composition of the thin films, we employed TEM on lamellae cut perpendicular to the film surface using FIB. Figure 3a–c presents a cross-sectional view of the Fe_4_N lamella. Overview image (Figure 3a) shows the stack of layers grown onto MgO substrate with a Fe_4_N film thickness of about 487(3) nm. Higher magnification image (Figure 3b) reveals the ∼13(2) nm Al capping layer. Perpendicular structural bands within the Fe_4_N film can be identified in Figure 3c. Selected area electron diffraction patterns along the [001] zone axis are shown in Figure 3d. It reveals the cubic symmetry, and the calculated lattice parameter *a* = 3.79 Å agrees well with the value obtained from XRD (3.80 Å). Furthermore, the diffraction pattern taken at the MgO substrate/Fe_4_N film interface region shows epitaxial coherent growth, confirming the XRD results, where only *h*00 reflections are detected.

**Figure 3:**
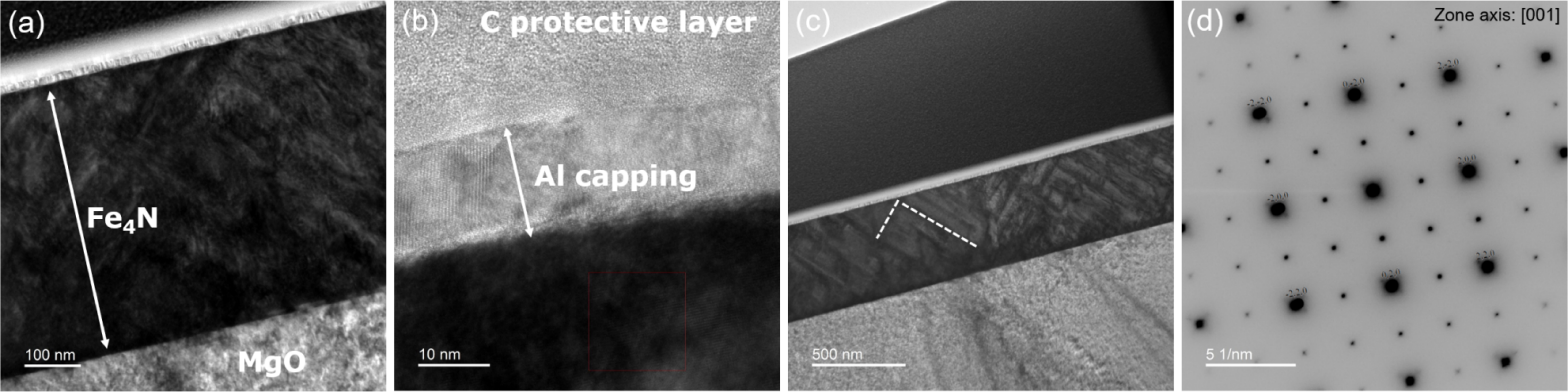
Transmission electron microscopy results for the Fe_4_N (*x* = 0) sample. (a) Overview image showing Fe_4_N thickness of about 487 nm. (b) Higher-magnification image reveals a 13 nm Al capping layer. (c) Perpendicular structural bands are observed in the Fe_4_N film. (d) Selected area electron diffraction pattern.

All three samples show band-like formations appearing as regions with varying contrast in the cross-sectional TEM. This effect is particularly illustrative in Fe_3_GeN shown in Figure 4. In Figure 4a, a low-magnification bright-field image of the thin film is pictured. Figure 4b shows the electron diffraction pattern of the thin film, and it shows two different lattice parameters for the tetragonal phase with lattice spacings of approximately 3.8 and 3.7 Å corresponding to the crystallographic *c* and *a* axes (the second significant digit cannot be extracted from the TEM data because of the experimental error). Using one of the diffraction spots (encircled), a dark-field image is produced and is shown in Figure 4c. It reveals distinct contrast differences and separation between these regions, suggesting a crystallographic orientation/distortion variation in the Fe_3_GeN film. The band-like features are parallel to each other and oriented 45° with respect to the film normal. These structural deviations indicate the coexistence of two different crystallographic orientations of the tetragonal phase where parts of the film have *a*-axis orthogonal to the surface in agreement with the XRD results summarized in [Table tbl1]. The two regions are separated by antiphase boundaries, as evident from the high-resolution TEM image shown in Figure 4d. To investigate the compositional differences, line and point energy-dispersive X-ray spectroscopy (EDX) was performed on these regions. However, no statistically significant variation in Fe and Ge contents could be detected, with the average chemical composition of around 74.4 at % Fe and 25.6 at % Ge. This suggests that the observed contrast differences may be related to variations in nitrogen content. Another possible explanation is that the band-like regions change with depth, and since TEM-EDX integrates data over the entire sample thickness, the average composition appears uniform across different areas. To further investigate this phenomenon, APT measurements were conducted and are described in the next section.

**Figure 4:**
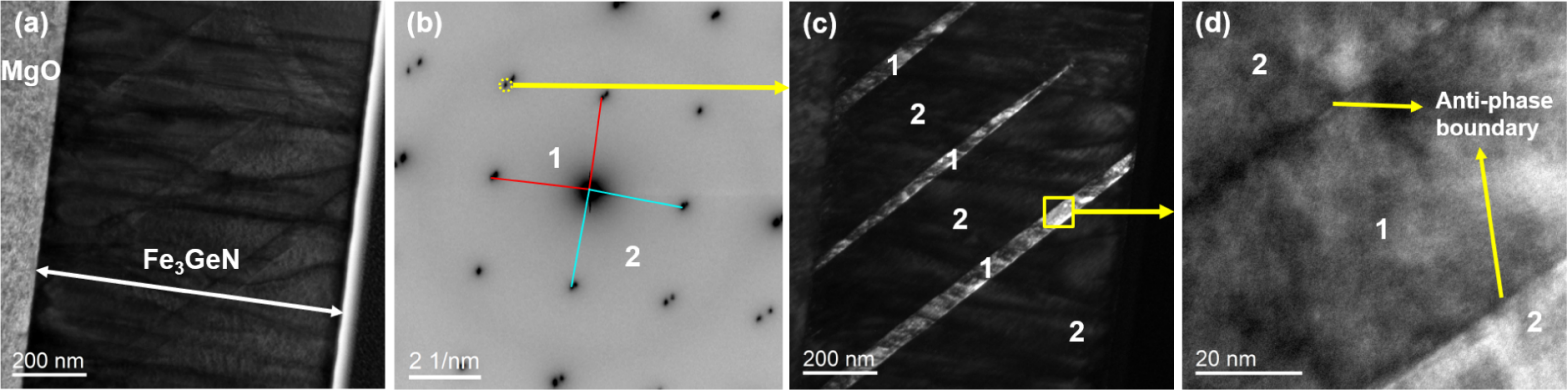
Transmission electron microscopy results for the Fe_3_GeN (*x* = 1) sample. (a) Overview low magnification bright-field image. (b) Electron diffraction pattern. (c) Dark-field image produced from the diffraction spot illustrated in (b). (d) High-resolution TEM image.

### Composition Variations at Band-Like Regions, *x* = 0, 0.5

APT enables spatially resolved analysis of the chemical composition at the nanometer scale in three dimensions and, therefore, was applied to investigate the micro/nano-structural features observed in the TEM. It has to be noted for proper interpretation of the results that the Fe–N system exhibits peak overlaps at 28 Da of Fe^2+^ and N_2_^2+^. Based on the detected isotopes, the peak at 28 Da has been assigned to Fe^2+^, and thus, the N content from APT is underestimated. An additional peak overlap of FeN^2+^ and Ge^2+^ is present in the Fe–Ge–N system at 35 Da. This peak has been assigned to Ge^2+^ based on the isotope ratios, and therefore, the Ge content is overestimated. Figure 5 presents APT results for the Fe_4_N and Fe_3.5_Ge_0.5_N samples. In the case of Fe_4_N, the Fe distribution appears uniform throughout the specimen, whereas N shows localized enrichment in band-like regions oblique to the film–substrate interface, which resembles the TEM observations discussed in the previous section. Figure 5b displays the compositional profiles for Fe and N integrated across the cylindrical volume sketched in Figure 5a. The cylinder has a diameter of 10 nm, and all atoms within the cylinder are counted for 1 nm slices. As explained above, the nitrogen content is consistently underestimated, showing values around 10 at %, although the actual concentration is expected to be closer to 20 at %. Nevertheless, there appears to be a slight reduction in Fe concentration and an increase in N across the band-like features. In the case of the Fe_3.5_Ge_0.5_N sample shown in Figure 5c, similar 45° inclined bands are observed. However, an interesting point to note is that in contrast to the Fe_4_N, they are formed by the change in composition of Fe and Ge and not due to a change in nitrogen content, which remains relatively constant throughout the analyzed cylindrical volume, see Figure 5d.

**Figure 5:**
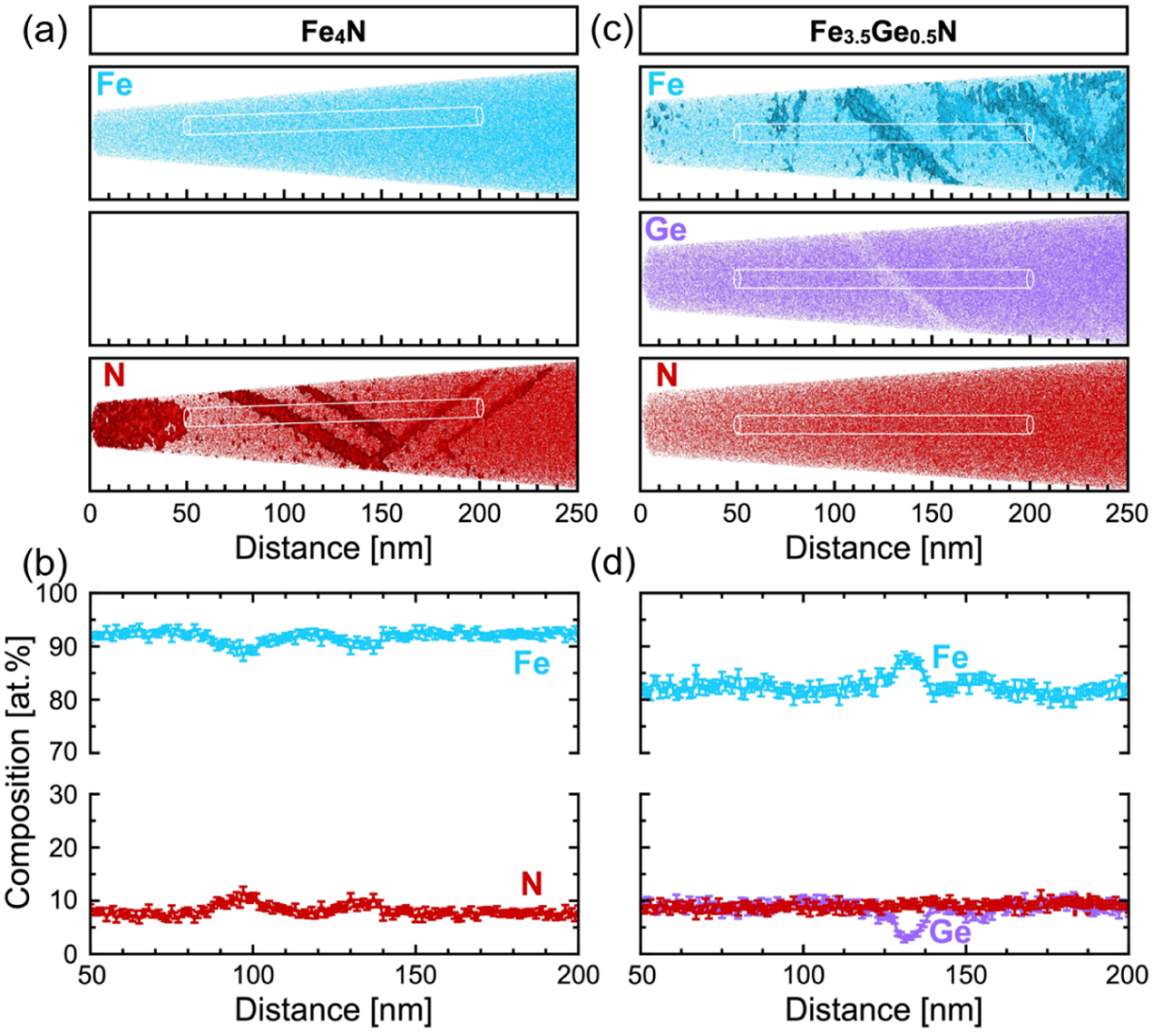
APT reconstructions for the Fe_4–*x*_Ge*_x_* N samples with (a) *x* = 0 and (c) *x* = 0.5. The respective compositional profiles for Fe, Ge, and N integrated across a cylindrical volume sketched in cylinders a and c with a diameter of 10 nm are shown in (b, d).

In summary of the experimental methods addressing the micro- and nanostructures: Using XRD, both *a*- and *c*-axis crystallographic orientations of the tetragonal *x* = 0.5 and *x* = 1 films were identified, while the fraction of *c*-axis orientation increases with *x*. The top-view SEM picture revealed stripe-like features for *x* = 1, and EDX determined different Ge contents in and away from the stripes, which can be qualitatively based on strain energy. The side-view TEM picture revealed similar perpendicular stripes (bands) inclined 45° toward the film surface; they may be ascribed to different crystallographic orientations as well. APT determined a lower Ge content in these stripes consistently with top-view stripes seen by SEM.

### Magnetic Properties

The magnetic moments calculated by DFT for Fe_4_N are 2.33 μ_B_ for Fe1 bonded to N and 2.93 μ_B_ for Fe2 in the large perovskite site, and the total calculated moment is 9.9 μ_B_ per formula unit (f.u.). This is higher than the experimental value of ∼8.5 μ_B_ obtained at 10 K (see Figure 6a). In our DFT calculations of tetragonal Fe_3_GeN, Fe1 crystallographic site is split into two Fe1c sites along *c*-axis and four Fe1ab sites in the *ab*-plane and Fe2 atom is replaced by Ge in accordance with the results of Mössbauer spectroscopy (see the Supporting Information) and refs 32, 33 . The total magnetic moment decreases to 2.3 μ_B_, not only because the magnetic Fe is replaced by Ge but also because the magnetic moment of the remaining Fe decreases, namely, Fe1c to 1.7 μ_B_ and Fe1ab even to 0.3 μ_B_, see also density of states (DOS) in Figure S2 in the Supporting Information. The experimentally determined value of the total moment ∼2 μ_B_ is somewhat lower than the calculated value (see Figures 7 and 10a). The critical temperature *T*_*C*_ decreases from 750 K for *x* = 0, to 550 K for *x* = 0.5, and to 100 K for *x* = 1, see Figure 7. The magnetic moment at 0.5 T decreases similarly from 7.8 μ_B_/f.u. for *x* = 0, to 3.3 μ_B_/f.u. for *x* = 0.5, and to 1.7 μ_B_/f.u. for *x* = 1. For *x* = 0 and *x* = 1, the shape of the *M*(*T*) loop is similar to conventional magnets, when *M* decreases quickly close to *T*_*C*_. In contrast, *M* decreases roughly linearly with increasing temperature for *x* = 0.5, which may be related to the disorder in the structure at the Fe2 site, where Fe and Ge alternate randomly, as opposed to *x* = 0 and 1, where the Fe2 position is fully occupied by Fe or mostly by Ge.

**Figure 6:**
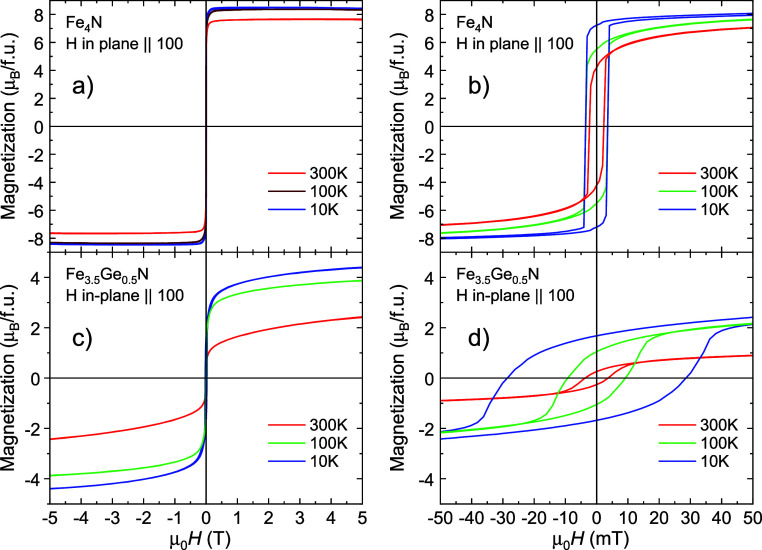
Hysteresis loops for (a) *x* = 0 and (c) *x* = 0.5, in-plane field. Right panels (b) and (d) display the detailed hysteresis near zero field.

**Figure 7:**
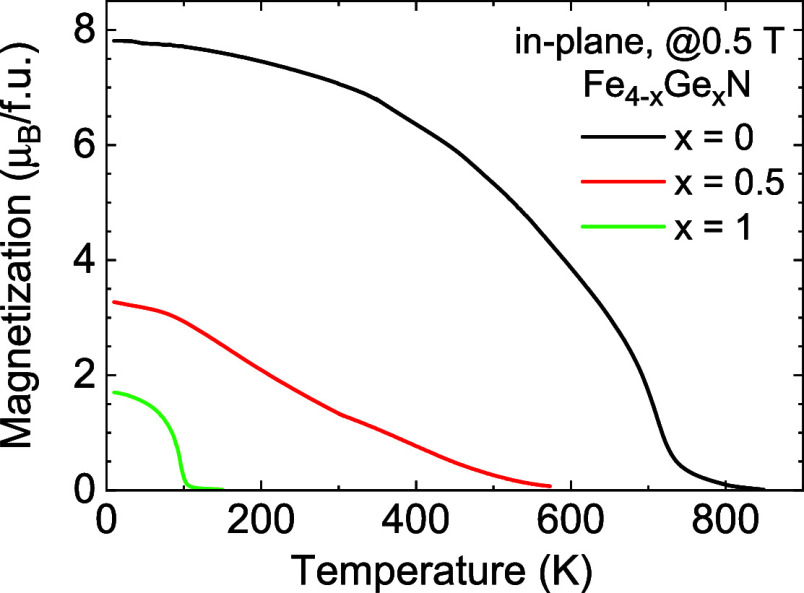
Temperature dependence of nearly saturated magnetization for Fe_4–*x*_Ge*_x_* N with *x* = 0, 0.5, and 1. Adding Ge drastically reduces both magnetic moment and *T*_*C*_, and for noninteger *x* = 0.5, the shape of the curve is distinct from *x* = 0 and *x* = 1 curves. Note that the low-temperature (<350 K) and high-temperature (>350 K) data were measured at different setups.

The in-plane hysteresis loops for *x* = 0 are displayed in Figure 6a,c and for *x* = 0.5 in Figure 6b,d. For *x* = 0, the curves are almost rectangular with remanent magnetization close to the saturated value, which can be ascribed to perfect crystallinity. For *x* = 0.5, the shape of the *M*(*H*) curves is more gradual. The higher coercivity in *x* = 0.5 can be due to either an increase in magnetocrystalline anisotropy caused by the introduction of Ge or random occupation of the site by Fe and Ge. For *x* = 1, the anisotropy decreases, which may nevertheless be caused by a decrease of magnetic interactions overall. The interesting shape of *M*(*H*) curves is for *x* = 1, for which we display both the in-plane and out-of-plane curves in Figure 10, together with Hall effect loops. In both cases, there is a two-knee feature, while the fields of the knees are shifted for the out-of-plane curves due to demagnetizing effects. The interpretation is discussed in the next section, together with transport measurements.

### Resistivity

The temperature dependence of resistivity for *x* = 0, 0.5, and 1 is displayed in Figure 8. At low temperatures, the temperature dependence of resistivity can be fitted by the relation ρ_0_ + *AT* ^*n*^ with *n* = 2.0 for all *x*. This type of dependence can be related to electron–electron scattering. The residual resistivity ρ_0_ is 3.1, 82, and 40 μΩ cm for *x* = 0, 0.5, and 1, respectively. A high residual–resistivity ratio (RRR) of the *x* = 0 sample, in our case calculated as *R*(300 K)/*R*(5 K) ∼ 27, indicates good crystallinity of the thin film. Other literature resistivity data of the Fe_4_N thin films show a smaller RRR within the range 3–10.^17,41–44^ Linear dependence (*n* = 1) is expected at a higher temperature; however, we observe an unusual sublinear (*n* = 0.33) temperature dependence, which cannot be explained by usual terms such as electron–electron, electron–phonon, or electron–magnon interactions. Nevertheless, the same sublinear dependence was also observed for Fe_4_N thin films by other authors.^17,41–44^ One explanation might be related to crystallographic defects connected with the lattice mismatch between Fe_4_N and MgO. However, the same behavior is observed for thin films deposited on LaAlO_3_ and SrTiO_3_, for which the mismatch is much smaller.^43^ Similar sublinear dependence was observed in other magnetically ordered metallic phases, e.g., SrIrO_3_,^45^ PdCrO_2_,^46^ or Ni_3_Sn_2_ ^47^ above the temperature of magnetic ordering and attributed to the gradual development of short-range spin correlations, which may reduce the magnetic scattering of the conduction electrons. However, in contrast to these references, in our case, we observe the phenomenon in the magnetically ordered state. Nevertheless, we propose that in our case, the magnetically ordered state can be perturbed by spin fluctuations that are induced by local magnetic moments associated with *N* vacancies. For this purpose, we have calculated the electronic structure of a 3 × 3 × 3 supercell with one empty *N* site to simulate *N* vacancy. This corresponds to Fe_4_N_0.96_ stoichiometry, comparable to that determined by EDX. The calculation revealed an enhancement of the magnetic moment for the Fe1 in the vicinity of the *N* vacancy up to 2.5 μ_B_, compared to Fe1 bonded to *N* having a magnetic moment 2.33 μ_B_. We propose that the random distribution of *N* vacancies and the associated random distribution of larger magnetic moments may result in a temperature dependence of the resistivity similar to that induced by spin fluctuations.

**Figure 8:**
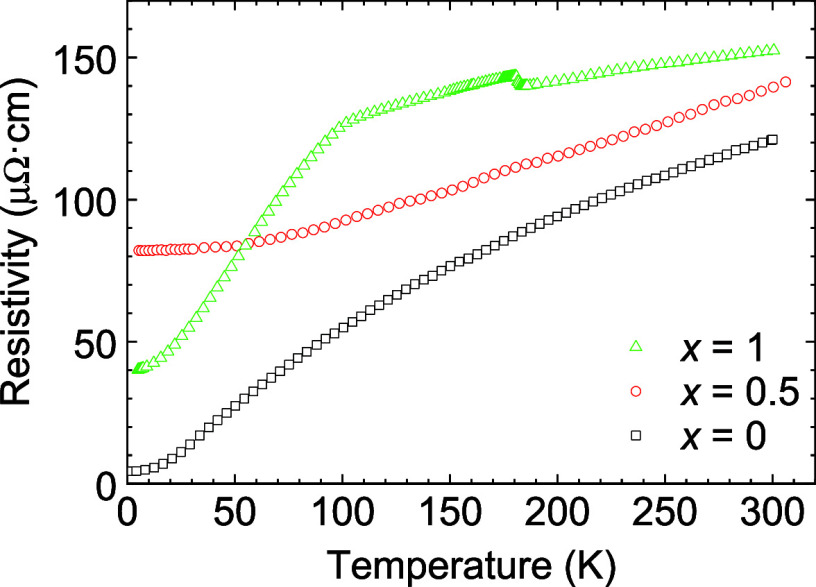
Temperature dependence of resistivity for Fe_4–*x*_Ge*_x_* N thin films with *x* = 0, 0.5, and 1.

The RRR ratio *R*(300 K)/*R*(5 K) is 4 for *x* = 1. The lower RRR is caused by two factors, namely, the higher residual resistance of 40 μΩ cm and the change in slope of the dependence at higher temperatures. The temperature dependence of resistivity shows two anomalies: a change of slope around 100 K connected with ferromagnetic transition *T*_*C*_, and a step down around 180 K, which we attributed to a small amount of secondary phase with a lower content of Ge. The sublinear resistivity dependence is observed in the temperature range just below *T*_*C*_ with *n* = 0.4, whereas above *T*_*C*_ the dependence on temperature is roughly linear. For *x* = 0.5, the RRR ratio is 1.8, which is the smallest value of the series. We relate the low RRR to the disorder in the structure at the Fe2 site (the large position of the antiperovskite structure), where Fe and Ge alternate randomly, as opposed to *x* = 0 and 1, where the Fe2 position is fully occupied by Fe or mostly by Ge. The sublinear behavior is not evident for *x* = 0.5, but it is probably masked by enhanced resistivity due to site disorder.

### Hall Effect, *x* = 0, 0.5

The temperature dependences of ordinary and anomalous Hall and Nernst effects extracted from the experimental hysteresis loops at individual temperatures are displayed in Figure 9 for *x* = 0, 0.5, 0.8, and 1. The measurement geometry for the Hall effect is displayed in Figure 11a and for the Nernst effect in Figure 11c. Nernst effect measurement using PPMS (Figure 11b) was only made for a few selected temperatures to determine the temperature gradient.

**Figure 9:**
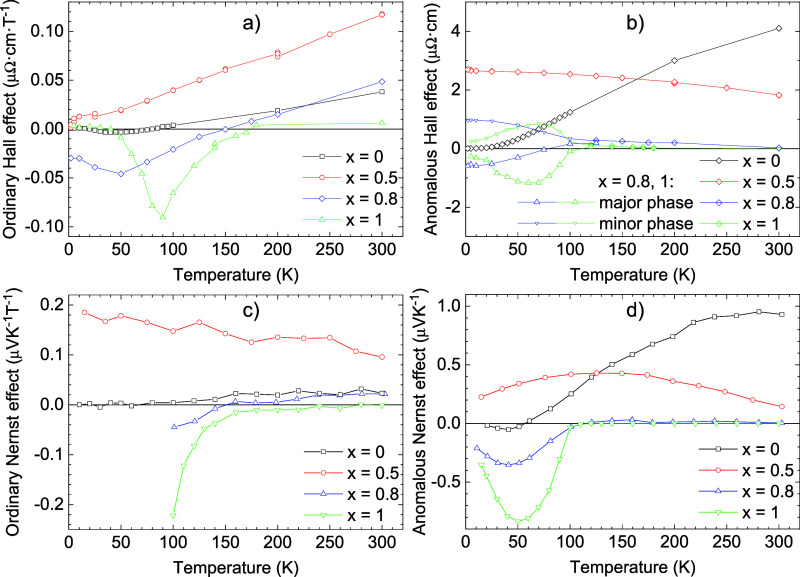
Temperature dependence of (a) ordinary Hall effect, (b) anomalous Hall effect, (c) ordinary Nernst effect, and (d) anomalous Nernst effect, for Fe_4–*x*_Ge*_x_* N with *x* = 0, 0.5, 0.8, and 1.

Ordinary Hall effect (OHE) for *x* = 0 is positive at room temperature, the dependence on temperature is linear, and it smoothly changes sign from positive to negative crossing zero at around 75 K, and then it changes back to a positive value around 20 K. This behavior reflects the properties of a multicarrier system, possessing a complicated band structure, where we can identify five bands crossing the Fermi level, see Figure 3 in ref 24. The bands have both hole and electron characteristics (negative and positive effective mass, respectively). The positive sign of the OHE indicates that the holes dominate the transport properties. The BoltzTraP2 calculation determined a positive Hall sign, in accordance with the experiment, but failed to reproduce the sign change at low temperatures. BoltzTraP2 uses the calculated band structure, so its output captures the number of carriers and their effective masses for all bands. However, its critical approximation lies in a single scattering time for all carrier types. We estimate that the sign change is related to the different temperature dependences of the scattering times of individual carriers. OHE for *x* = 0.5 is also positive at room temperature and linearly decreases toward zero at low temperatures. A higher value of the OHE compared to *x* = 0 indicates a lower apparent carrier concentration. Anomalous Hall effect (AHE) for *x* = 0 is also positive at room temperature, decreases almost linearly with decreasing temperature, and tends toward zero at the lowest temperature. In contrast to OHE, no change of sign is observed.

A relation linking anomalous Hall resistivity $\rho_{xy}^{AH}$ and longitudinal resistivity ρ*_xx_* has been devised in the form

2 $$\rho_{xy}^{AH}∼\lambda \rho_{xx}^{n}+\rho_{0}^{\prime }$$

where *n* and λ are nondimensional exponent and prefactor respectively,^48^ and $\rho_{0}^{\prime }$ is for separating the residual resistance.^17^ The exponent *n* in this power-law relation is related to different mechanisms of the AHE. The intrinsic models that only depend on the ideal band structure and are independent of scattering include interband effect and the Berry curvature mechanisms. The models based on extrinsic mechanisms include side jump and skew scattering. The intrinsic and side jump mechanisms can be modeled with the exponent *n* = 2, while the skew-scattering mechanism predicts *n* = 1. The scaling relation ([Disp-formula eq2]) was intensively investigated in ref 17 on a series of epitaxial and polycrystalline Fe_4_N thin films with varying thickness. The exponent was determined close to *n* ∼ 2 for epitaxial films but smaller than 2 for polycrystalline films. The authors focused on data from the temperature range below 125 K, as they observed a deviation from the fit for higher temperatures, which they explained by a decrease in AHE associated with decreasing magnetization. Similarly, in our case of epitaxial thin films, we obtained good agreement of the fit with an exponent *n* = 1.94 up to a temperature of 100 K, while adequate fit cannot be achieved when a wider temperature range is used, see Figure S3 in the Supporting Information. The obtained exponent close to *n* ∼ 2 is consistent with the assumption that the dominant mechanism of AHE and ANE can be explained by an intrinsic model including the Berry curvature.

### Nernst Effect, *x* = 0, 0.5

The Nernst effect was measured using the homemade apparatus, where the magnetic field and the measured electric field are in the plane of the thin film and the temperature gradient is perpendicular to the thin film; see Figure 11c. The Nernst effect loops for *x* = 0, 0.5, and 1 are shown in Figure S4 and in the Supporting Information. The hysteresis loops are comparable with in-plane magnetic measurements. In this configuration, the temperature gradient cannot be measured separately for the thin film and the substrate, but the sum of both contributions is determined. The total gradient is divided proportionally according to the thermal resistances of the film and substrate. However, the thermal resistance of the materials involved may not be known or may be different than the literature values for the specific samples used. Therefore, the determination of the thermal gradient of the film itself is uncertain. A more accurate way is to make additional measurements using the configuration shown in Figure 11b, which is available in the PPMS apparatus. In this case, the temperature gradient is applied in the plane of the film and can be accurately determined. Since the temperature evolution of the thermal resistances of the film and substrate is generally different, several selected temperatures were measured. The hysteresis loops are comparable with out-of-plane magnetic measurements. In agreement with the Berry curvature calculation (see Figure 12), the ANE for *x* = 0 is positive. The maximum at room temperature is 0.9 μV/K. It decreases by lowering the temperature and changes the sign around 55 K, and after a minimum around 40 K tends to zero at the lowest temperature. In the case of *x* = 0.5, ANE is positive with a maximum around 100 K and tends to have a positive value at the lowest temperature.

### Hall and Nernst Effects, *x* = 1

Magnetization loops for *x* = 1 exhibit a two-component character, see Figure 10. The in-plane loop (Figure 10a) grows quickly to an almost saturated value in the low-field region, followed by a slow linear increase, saturating at ∼2 T. The out-of-plane curve (Figure 10b) exhibits a similar behavior, but the contribution of the slowly saturating part to the total moment is much larger. Note that magnetization still increases above 2 T, which is ascribed to a frustrated ferromagnetic state.^28,29^ The two-component feature is more prominent in the Hall effect measurements (Figure 10c), corresponding to the out-of-plane setup (Figure 11a), because the two components have opposite AHE signs. The example of decomposition of the loop into the contribution of the OHE and two contributions of the AHE for *T* = 40 K is displayed in Figure 11a. A similar shape with two ANE contributions of opposite signs is also observed for Nernst effect hysteresis loops; see analysis in Figure 11b.

**Figure 10:**
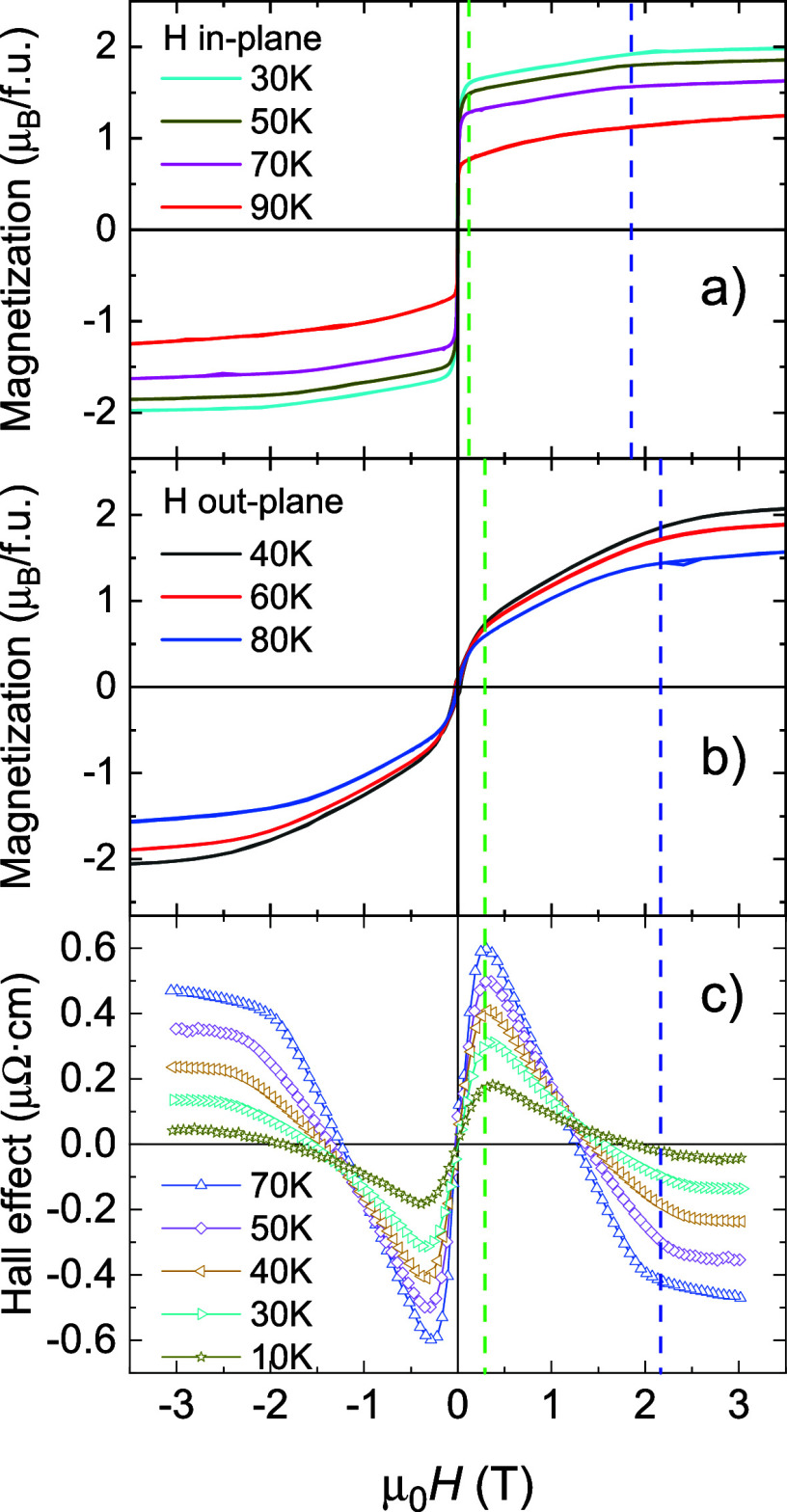
(a) In-plane and (b) out-of-plane magnetization curves for Fe_3_GeN as compared to (c) Hall effect loops. Both in-plane and out-of-plane magnetization curves show 2-step behavior. Critical fields are higher for the out-of-plane case due to the demagnetization field. Hall effect loops correspond to the out-of-plane configuration, and the two-step feature is even more prominent, because the two contributions have opposite signs of AHE, corresponding to different critical fields.

**Figure 11:**
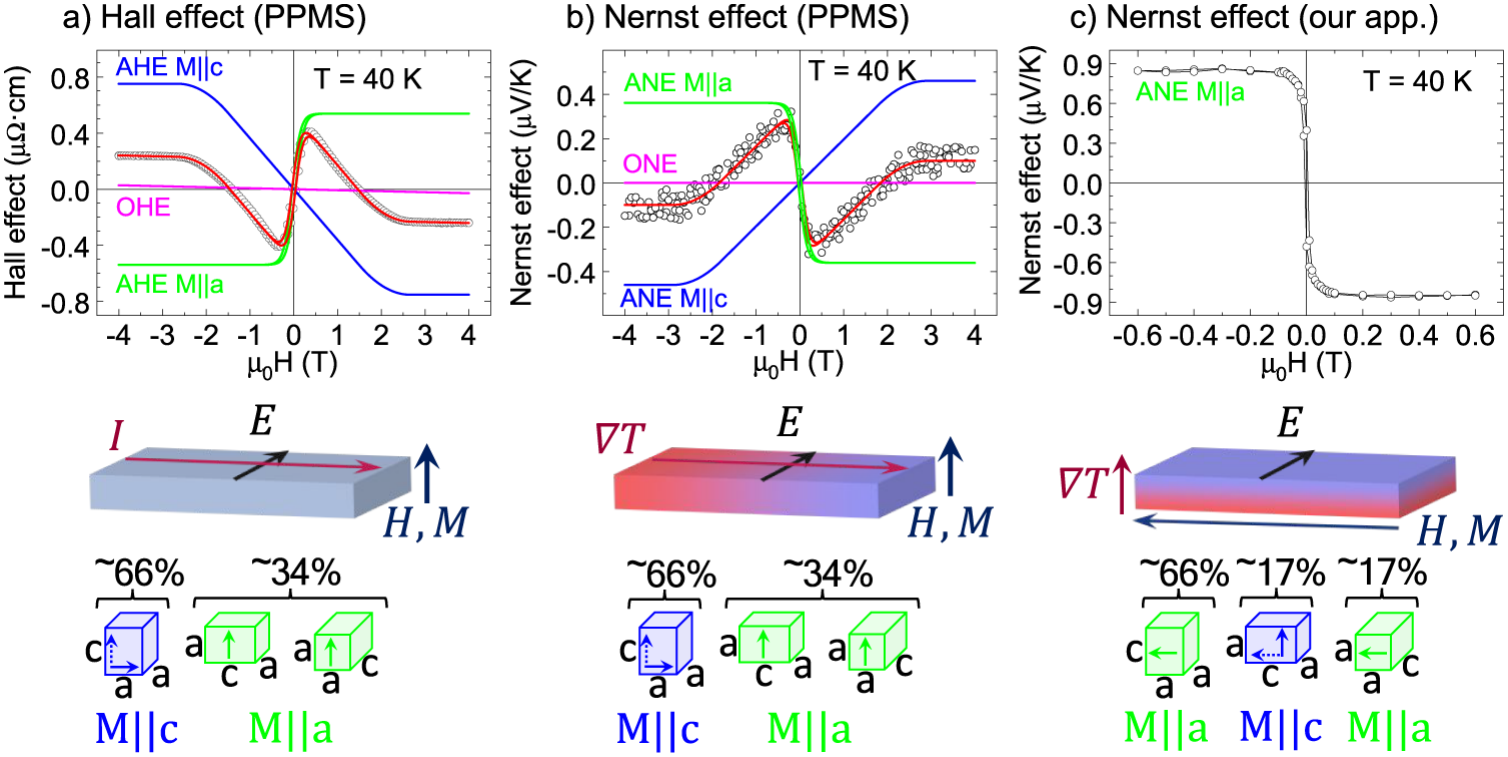
Hall and Nernst effect loops for tetragonal Fe_3_GeN at *T* = 40 K with simulation of two-component AHE/ANE and linear OHE/ONE. Data and measurement geometry for (a) Hall effect in PPMS apparatus, (b) Nernst effect in PPMS, and (c) Nernst effect in homemade apparatus. Black circles: experimental data (corrected for constant and symmetric contributions). Magenta lines: OHE or ONE component. Blue curves: AHE or ANE component for magnetization parallel to the *c*-axis. Green curves: AHE or ANE component for magnetization parallel to *a*-axis (see corresponding calculations in Figure 12). Red curves: sum of all contributions, fitting the experimental data. Displayed tetragonal unit cells show the distribution of crystallographic orientation in the film as determined by XRD. Full arrows in the unit cells indicate magnetization in the direction of the easy *a*-axis, and dotted arrows denote magnetization rotated to the direction of the hard *c*-axis.

A similar two-component character of the Hall effect was observed in ref 49, where the authors reported the observation of AHE in (Bi,Mn)_2_Se_3_ thin films and showed that the sign of AHE changes from positive to negative as the Mn concentration is increased. The positive and negative AHE were found to coexist in a crossover regime and could be distinguished due to significantly different saturation fields. The authors assigned positive AHE with a higher saturation field to the bulk states and negative AHE with a lower saturation field to the surface states.

In our case, we propose that the two-component shape of the Hall and Nernst loops is caused by the different signs of Hall and Nernst effects of the majority and minority phases forming the dark stripes observed by SEM, see Figure 2. First, we tested the possibility that the behavior is related to the lower Ge content of the stripes. For this purpose, we have prepared thin films with *x* = 0.8 and measured Hall and Nernst effects. In the case of this scenario, we would see opposite signs of the Hall effect for *x* = 0.8 and *x* = 1. However, we also observed a similar two-component behavior for *x* = 0.8, but with different temperature dependences, see Figure 9b. We then focused on the possibility that the interpretation is related to distinct AHE and ANE signs along different crystallographic directions of the Fe_3_GeN tetragonal cell. For this purpose, we have calculated anomalous Hall and Nernst conductivities using DFT and the Berry curvature mechanism. The calculations indeed confirmed opposite signs of the effects calculated for magnetization *M* along [100] and [001] directions, both for AHC and ANC, see Figure 12.

**Figure 12:**
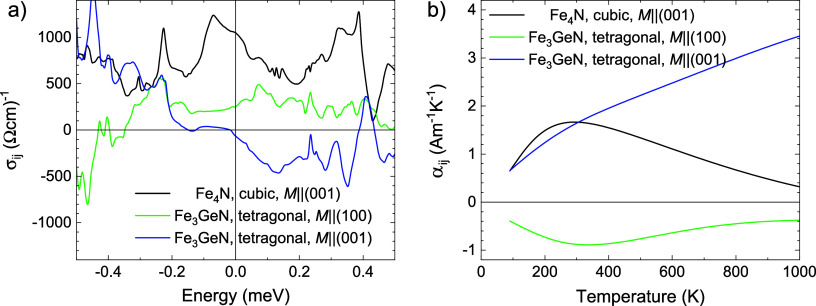
Comparison of the calculated intrinsic (a) AHC (σ_*ij*_) and (b) ANC (α*_ij_*), for cubic Fe_4_N and tetragonal Fe_3_GeN for magnetization along *c*-axis *M*||[001] and along *a*-axis *M*||[100]. Calculation was made using the Berry curvature mechanism.

Therefore, we propose that the two-component shape of the hysteresis loops is related to the two orientations of tetragonal cells in thin films (see the XRD and SEM sections) and opposite signs of the anomalous Hall and Nernst effect for magnetization along the *c* and *a*-directions. According to XRD, the majority (∼80%) of the *x* = 1 thin film is oriented with the *c*-axis perpendicular to the surface. For the following analysis, we prefer the use of the ratios of crystallographic orientations determined from magnetization curves 66:34, see Figure S7 and modeling magnetism for Fe_3_GeN section in the Supporting Information, which is supposed to be more accurate than from XRD (80:20), which relies on the relative analysis of few diffraction peaks, whose intensities may be affected by different degrees of preference orientation in the epitaxial thin films, while magnetism reflects integral information. When measuring the Hall effect, the electric current and the measured voltage are in the plane; see Figure 11a. This means that AHE is observed for the magnetization direction along [001], which, according to Berry curvature calculations, should be negative; see Figure 12a. The minor part of the thin film is oriented with *c*-axis parallel to the surface, so for this part, the AHE with magnetization along [100] or [010] is detected, which is positive, see Figure 12a. This explanation is also supported by the measurement of the Nernst effect for *x* = 1 using PPMS, which is measured in the same geometry; just the electric current is replaced by a temperature gradient. According to the Berry curvature calculations, the ANE measured with magnetization along the *c*-direction is positive and ANE measured with magnetization along the *a*-direction is negative, i.e., opposite signs compared to AHE, see Figure 12b. This is consistent with the experiment, since we also observe a two-component character of the hysteresis loop for the Nernst effect, but with opposite signs; see Figure 11b.

Measurements of the Nernst effect performed in the homemade apparatus differ in the experimental setup, namely in the interchange of directions of ∇*T* and *M*, and also in a lower attainable field, see Figure 11c. In this setup, the ANE ratios for *M*||*c* and *M*||*a* are substantially different. While for the PPMS setup, it should correspond to the ratio of crystallographic orientations of approximately 66:34, in the homemade apparatus, this ratio should be about 17:83 (assuming that for orientation with *a*-axis perpendicular to the surface, the portion of the *c*-axis orientation is the same for both directions). The negative value of ANE for *M*||*a* should therefore be approximately 2.5× greater, and the positive value for *M*||*c* approximately 4× smaller. Since the measured magnetic field range is smaller than the saturation field for *M*||*c*, the two-component behavior is not visible in this measurement, and the contribution of ANE with *M*||*c* only exhibits a slight linear decrease in the Nernst signal, which is essentially indistinguishable from the ordinary Nernst effect (ONE) contribution. Therefore, ONE contribution was not determined for *x* = 1 below *T*_*C*_ (Figure 9c). As regards the absolute value of the negative ANE determined by our apparatus, it is about 2.4× greater than in the PPMS setup, which is in very good agreement with the assumed ratio of crystallographic orientations.

In order to explain different saturation fields for the *a-* and *c*-directions, we have calculated the energy differences among various magnetization directions by DFT calculations including spin–orbit coupling. It revealed that the *M*||[100] configuration has lower energy by 0.272 meV/f.u. than *M*||[001]. This energy difference corresponds to an anisotropy field of 2.3 T, assuming a saturated magnetic moment of 2 μ_B_ for 40 K, see Figure 10. The observed saturation field of the unfavorably oriented component at around 2 T is in good agreement with the calculated energy difference. Moreover, the magnitudes of magnetization can also be explained within this scenario: For the in-plane measurement (Figure 10a), the majority orientation with large contribution is saturated at low fields, while for the out-of-plane case (Figure 10b), the minority orientation is saturated at low fields, and majority of the signal is gained between 0.3 and 2.2 T (between two dashed lines in Figure 10b). Analysis of 2-component magnetization curves determined 66% of *c*-axis-oriented domains and ∼34% of *a*-oriented domains, see Figure S7 in the Supporting Information.

Altogether, we ascribe the two-component anomalous Hall and Nernst effects to different crystallographic orientations exhibiting effects with opposite signs and having different saturation fields due to magnetic anisotropy. Our argumentation is as follows:

1 The presence of both *c*-axis and *a*-axis crystallographic orientations is revealed by XRD measurements (Figure S1 in the Supporting Information).
2 Opposite signs of the anomalous Hall and Nernst effects for different crystallographic orientations (Figure 11) have been confirmed by DFT and Berry curvature calculations (Figure 12).
3 Moreover, the DFT results also predict quantitatively well the magnetic anisotropy in Fe_3_GeN, which is responsible for different saturation fields. DFT is supposed to evaluate the magnetic anisotropy much better than it evaluates the Nernst and Hall effects, so its reliability is supposed to be very high in this case. Another convincing argument is the magnetization curves (Figure 10a,b), showing the two-component behavior. Both saturation fields and magnetization steps are consistent with the Hall, Nernst (Figure 10c, Figure 11) and XRD data.

## Conclusions

A series of Fe_4‑x_Ge_*x*_N (*x* = 0–1) thin films were grown onto MgO substrates by magnetron sputtering. All the deposited thin films exhibit epitaxial growth. The Fe_4_N (*x* = 0) phase crystallizes in the cubic *Pm*3̅*m* space group, whereas a small crystallographic tetragonal distortion toward *I*4/*mcm* symmetry is realized in Fe_4–*x*_Ge_*x*_N films above *x* ∼ 0.35. From a comparison of Mössbauer spectroscopy and DFT calculation results, we conclude that Ge occupies the 4*b* site in the tetragonal structure. All samples are ferromagnetic, with Curie temperature decreasing rapidly from 750 K for *x* = 0 to 100 K for *x* = 1.

An unusual sublinear temperature dependence of resistivity was observed for *x* = 0 and 1, which cannot be explained by standard electron interactions. A similar type of dependence was identified for some magnetically ordered metallic phases, where it was attributed to the gradual development of short-range spin correlations above *T*_*C*_. We propose that in our case, where the sublinear dependence is observed below *T*_*C*_, the random distribution of *N* vacancies and the associated random distribution of larger magnetic moments perturb the magnetically ordered state, which may result in a similar temperature dependence of the resistivity as that induced by spin fluctuations. The unusual behavior was not clear for the *x* = 0.5 sample, differing in several aspects from *x* = 0 and *x* = 1, which was ascribed to compositional disorder. Besides resistivity, for which the *x* = 0.5 sample showed the lowest RRR, the anomalies were observed in magnetization and mechanical measurements (see the Supporting Information) compared to *x* = 0 and *x* = 1 samples.

The anomalous Hall and Nernst effects are positive for *x* = 0 and 0.5. The maximum ANE signal was determined to be 0.9 μV/K for *x* = 0 at room temperature. We achieved a good fit between the scaling relation $\rho_{xy}^{AH}∼\lambda \rho_{xx}^{n}+\rho_{0}^{\prime }$ and the data up to a temperature of 100 K, whereas sufficient agreement cannot be reached when using a wider temperature range. The obtained exponent *n* = 1.94 is consistent with the assumption that the dominant mechanism of AHE and ANE can be explained by an intrinsic model that also includes the Berry curvature, for which *n* = 2 is expected.

The maximum ANE signal for *x* = 1 is −0.85 μV/K at *T* = 50 K. The rapid increase of ANE of Fe_3_GeN from low temperatures indicates that it would surpass ANE of Fe_4_N if it were not limited by the low *T_C_*. This observation is consistent with our theoretical assumptions and encourages further attempts at syntheses of doped Fe_4_N, where ANE enhancement is expected according to DFT combined with Berry curvature calculations. The hysteresis loops of the Hall and Nernst effects for *x* = 1 exhibit unusual two-component shapes, which can be described as the sum of positive and negative loops with different saturation fields. The same two-component behavior is observed in magnetization loops. We explained this observation by the presence of two different crystallographic orientations in thin films, with the majority (minority) of the sample oriented with the *c-(a-)* axis perpendicular to the film surface, and opposite signs of the respective phenomena for different directions. The opposite signs of the effects were confirmed by DFT calculations in combination with the Berry curvature method. The presence of two different orientations was revealed by XRD and is consistent with the observation of stripes forming an almost regular orthogonal structure with varying Ge content by SEM, the detection of bands with different crystallographic orientation by TEM, as well as variations of Ge content in these bands determined by APT. The formation of these stripes is driven by the tendency to partially reduce the lattice mismatch between the film and the substrate.

## Supplementary Material



## Data Availability

Data associated with this study are available on the Zenodo repository at 10.5281/zenodo.18197298

## Notes

Additional experimental information, hardness, and Mössbauer measurements; additional figures of XRD, Hall and Nernst effects, and figures of DOS; and analysis of magnetic data (PDF)
