# Supplementary material for: Two-Component Anomalous Hall and Nernst Effects in Anisotropic Fe4– x Ge x N Thin Films
Source: ACS Omega. 2026 Jan 27;11(5):8748–60. doi: 10.1021/acsomega.5c12420 (PMC12903171; doi:10.1021/acsomega.5c12420)
Supplement: Supplementary file 1 [file ao5c12420_si_001.pdf]

Supporting information for:
Two-component anomalous Hall and Nernst effects in anisotropic $Fe_{4-x}Ge_xN$ thin films

Robin K. Paul [1], Jakub Vít [2], Petr Levinský [2], Jiří Hejtmánek [2], Ondřej Kaman [2], Mariia Pashchenko [2], Lenka Kubíčková [2], Kyo-Hoon Ahn [2], Markéta Jarošová [2], Joris More Chevalier [3], Stanislav Cichoň [3], Tomáš Kmječ [4], Jaroslav Kohout [4], Marcus Hans [5], Stanislav Mráz [5], Jochen M. Schneider [5], Esmaeil Adabifiroozjaei [6], Leopoldo Molina-Luna [6], Oliver Gutfleisch [1], Imants Dirba [1**], Karel Knížek [2*]

[1] Functional Materials, Institute of Materials Science, Technical University of Darmstadt, Peter-Grünberg-Str. 16, 64287 Darmstadt, Germany

[2] FZU – Institute of Physics of the CAS, Cukrovarnická 10, 162 00 Praha 6, Czech Republic

[3] FZU – Institute of Physics of the CAS, Na Slovance 1999/2, 182 00 Praha 8, Czech Republic

[4] Faculty of Mathematics and Physics, Charles University, V Holešovičkách 747/2, 180 00 Praha 8, Czech Republic

[5] Materials Chemistry, RWTH Aachen University, Kopernikusstr. 10, 52074 Aachen, Germany

[6] Advanced Electron Microscopy Division, Institute of Materials Science, Technical University of Darmstadt, Peter-Grünberg-Str. 22, Darmstadt 64287, Germany

Experimental

Thin lamellae of Fe_4N, $Fe_{3.5}Ge_{0.5}N$ and Fe_3GeN films for transmission electron microscopy (TEM) were prepared by focused ion beam (FIB) within a FEI Helios Nanolab 660 dual-beam microscope. The region of interest was covered with an Al protective layer of 8 µm length and 2 µm width. First ~50 nm of C were deposited with the electron beam, followed by ~1.5 µm of Pt using Ga ions. The lift-outs were attached to Omniprobe holders and thinning was done by milling a 4 µm wide window with a final lamella thickness of ~50 nm. High-resolution transmission electron microscopy was performed using a JEOL JEM 2100F with an acceleration voltage of 200 kV. ImageJ software was used for image processing and analysis. The interpretation of the selected-area electron diffraction images was done by the use of the CrysTBox [1].

The chemical composition of the inner region of the films was investigated using atom probe tomography (APT). This technique combines mass spectrometry as well as projection microscopy and is based on field evaporation [2]. A voltage is applied between a needle-shaped specimen and an electrode, while control of the field evaporation process is obtained by additional application of voltage or laser pulses. Atoms are eventually evaporated and ionized within the electric field. These ions are accelerated towards a position-sensitive detector, and a mass spectrum is obtained through a time-of-flight measurement. Based on the hit position at the detector, the original position of an atom within the specimen before field evaporation can be reconstructed using projection algorithms. This makes APT a powerful tool enabling identification of changes of the chemical composition at materials defects such as interfaces [3] or grain boundaries [4].

Needle-shaped specimens were fabricated by FIB techniques in the FEI Helios Nanolab 660 dual-beam microscope. Ga ions were employed at 30 kV and a standard protocol has been followed [5]. The specimen preparation was finished with a low voltage cleaning at 5 kV and 40 pA for 30 seconds. APT specimens were transferred to a CAMECA local electrode atom probe (LEAP) 4000X HR and the exposure time to atmosphere during transfer was < 3 min. Field evaporation was assisted by thermal pulsing, employing an ultraviolet laser. A relatively low pulse energy of 10 pJ was chosen in order to maximize the electric field strength and thereby enhance the measurement accuracy [6]. The laser pulse frequency was 200 kHz, while the base temperature of 60 K and detection rate of 0.5% were set.

Ranging of the mass spectra and reconstruction of the atomic positions has been carried out in the AP Suite 6.2 software package.

The mechanical properties were characterized by nanoindentation using a Hysitron TI-900 TriboIndenter. Quasistatic measurements were conducted with a 100 nm Berkovich diamond tip and the load-displacement curves were evaluated according to the method from Oliver and Pharr [7]. Load-controlled measurements were carried out with maximum load of 1500 µN, resulting in contact depths of 53 to 58 nm for Fe_4N, 45 to 54 nm for $Fe_{3.5}Ge_{0.5}N$ and 47 to 56 nm for Fe_3GeN. The tip area function was determined from a fused silica reference sample.

Room-temperature ^{57}Fe conversion-electron Mössbauer spectra of the $Fe_{4-x}Ge_xN$ samples with x = 0.5 and 1.0 were obtained in a constant-acceleration mode using a ^{57}Co/Rh source. The sample plane was perpendicular to the direction of the γ-rays. The velocity scale was calibrated against a room-temperature spectrum of an α-Fe foil, and isomer shifts are reported relative to its centroid. Spectral analysis was performed using the Confit software [8] (x = 1) or the relaxation model based on the work of Blume and Tjon [9] implemented in the Recoil [10] program (x = 0.5).

Results and Discussion

X-ray powder diffraction

Since the Fe atoms are arranged in an *F*-centered cell and the *F*-centering is only broken by weakly diffracting nitrogen atoms, the *h*00 reflections with even *h* (allowed for *F*-centering) are much stronger than the reflections with odd *h* (forbidden for *F*-centering). Within the diffraction range of CuKα radiation, the strong 200 and 400 and weak 100 reflections are visible for the cubic Fe_4N epitaxial thin films.

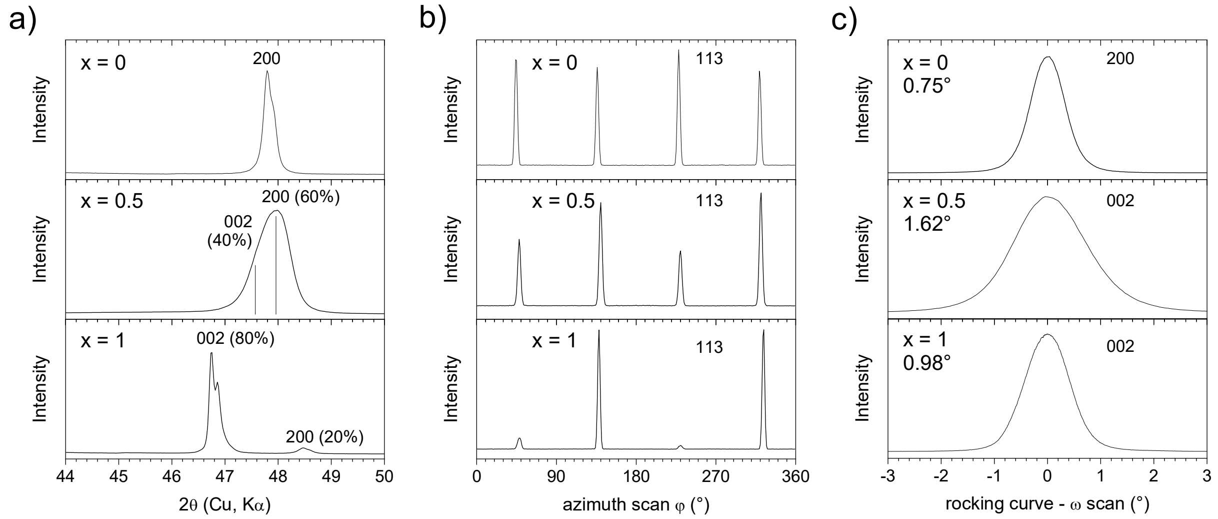

Figure S1. X-ray diffraction of $Fe_{4-x}Ge_xN$ thin films on MgO substrate. (a) Diffraction pattern of *200/002* reflection, (b) azimuthal scan of *113* reflection, (c): Rocking-curve scan of *200/002* reflection. *hkl* indexes are related to the cubic cell.

Indentation modulus and hardness

The results of indentation experiments show the same elastic Young modulus for x = 0 and 0.5 and a significant decrease in the modulus for x = 1. On the other hand, the hardness determined by this method exhibits an increase from x = 0 to x = 0.5 and then a slight decrease, but with x = 1 remaining harder than x = 0, see Table S1. For the confrontation of experimental values with calculation, we have performed the DFT electronic structure calculation of elastic properties. The parameters *C11*, *C12* and

C44 were evaluated for cubic structure of $x = 0$, and parameters *C11*, *C33*, *C12*, *C13*, *C44* and *C66* for the tetragonal structure of $x = 1$, where we supposed full occupation of the Fe2-site by Ge and the Fe1-site by Fe, as confirmed by Mössbauer results. Using these parameters, several elastic properties were calculated, including elastic wave velocity and Debye temperature, see Table S2. Hardness was calculated using models suggested in [11]. Generally, the calculated elastic quantities increased for $x = 1$. This trend could be expected, since Ge introduces more covalent character into the structure, so it increases the strength of the bonds [12]. The calculated hardness is in quite good agreement with experiment as regards the trend as well as the absolute values. They are also in good agreement with previous literature data of Fe_4N being consistently around 8 GPa [13,14].

On the other hand, the calculated elastic moduli show an opposite trend to the experiment. Possible explanations may be related to the lattice mismatch between the film and substrate, which increases with x, and the resulting defects in the thin film. Previously obtained experimental data on Young modulus of Fe_4N are also rather inconsistent, ranging from 160 to 210 GPa [13,14,15,16]. These differences in experimental data may be caused by different types of samples (thin layers, bulk samples, etc.), their microstructure and different methods used. The range of previous literature data thus includes both our experimental and calculated values for the elastic modulus of Fe_4N. Comparison with literature data therefore does not unambiguously distinguish which of these values is more accurate. Regarding Fe_3GeN, no literature data on elastic properties are available.

Table S1. Average values of Young modulus (*E*) and hardness (*H*) determined by indentation and by DFT calculation.

	E_{exp} (GPa)	H_{exp} (GPa)	E_{DFT} (GPa)	H_{DFT} (GPa)
Fe_4N	203 ± 8	8.1 ± 0.2	162	7.6
$Fe_{3.5}Ge_{0.5}N$	204 ± 5	9.5 ± 0.4		
Fe_3GeN	156 ± 4	8.8 ± 0.4	222	9.2

Table S2. Results of DFT calculation (Wien2k package) of elastic properties (IRelast package).

Mechanical properties	Fe_4N	Fe_3GeN
Bulk modulus, *K* (GPa)	193.91	227.14
Young modulus, *E* (GPa)	161.74	221.53
Shear modulus, *G* (GPa)	59.42	82.82
Poisson's coefficient, *n*	0.36	0.34
Hardness, *H* (GPa)	7.6	9.2
Zener elastic-anisotropy ratio, *A*	0.45	0.57
Pugh ratio, *k*	0.31	0.36
Transverse elastic wave velocity, v_t (m/s)	2861.85	3205.13
Longitudinal elastic wave velocity, v_l (m/s)	6135.70	6470.94
The average elastic wave velocity, v_m (m/s)	3222.40	3597.53
Debye Temperature, θ (K)	433.14	489.57
Stiffness tensor		
C11 (GPa)	320.25	388.49
C33 (GPa)		423.53
C12 (GPa)	130.74	148.77
C13 (GPa)		136.71
C44 (GPa)	43.08	59.99

C66 (GPa)		62.79

DFT calculations

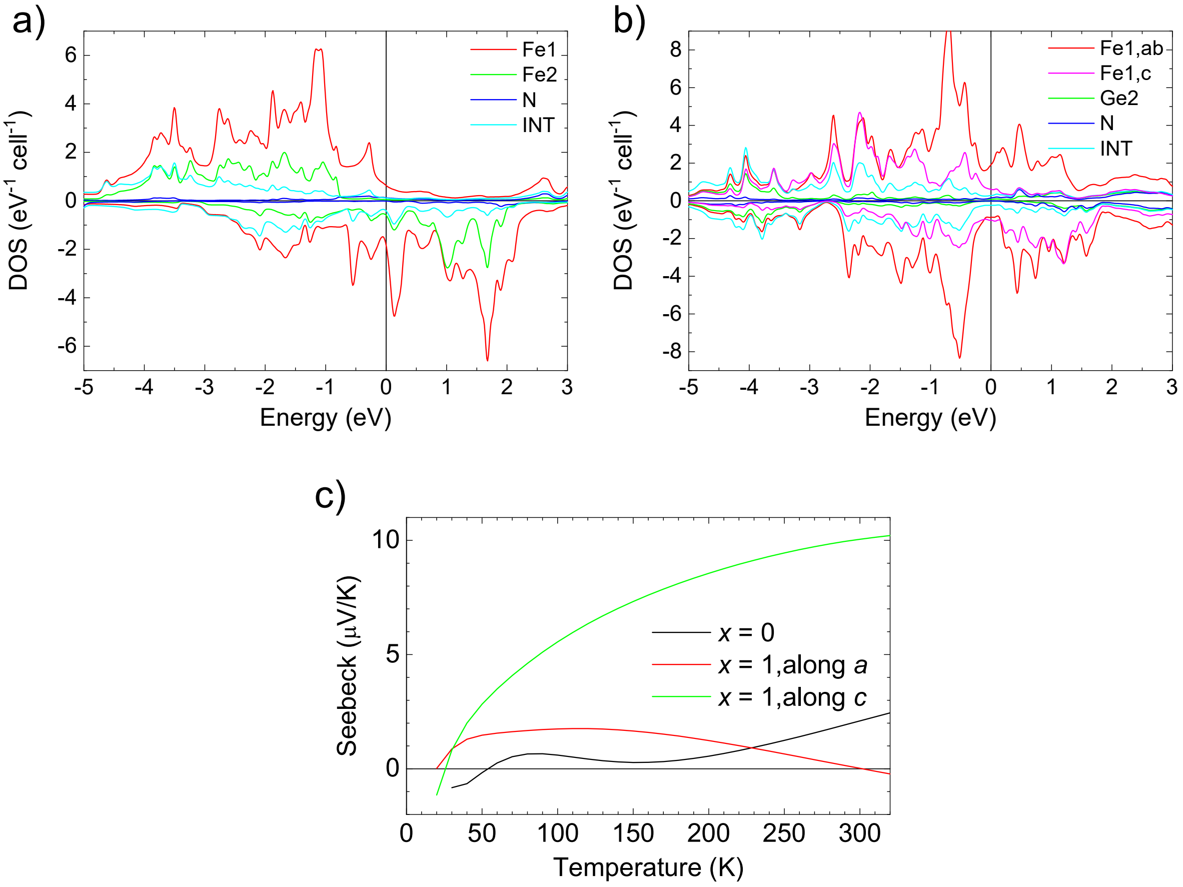

Figure S2. Density of states calculated for $Fe_{4-x}Ge_xN$ with (a) x = 0 and (b) x = 1. Partial DOS for individual atoms and for interstitial region (INT), which cannot be unambiguously assigned to a specific atom, are shown. Note the reduction of spin polarization for x = 1. (c) Seebeck coefficient calculated by BoltzTraP2 for cubic x = 0, and for tetragonal x = 1 along *a* and *c*-axis. Seebeck coefficient along a and c-direction for x = 1 shows quite a big difference as regard absolute value, so it confirms significant difference of transport properties between a and c, although the sign is the same (positive for both directions).

Hall and Nernst effect

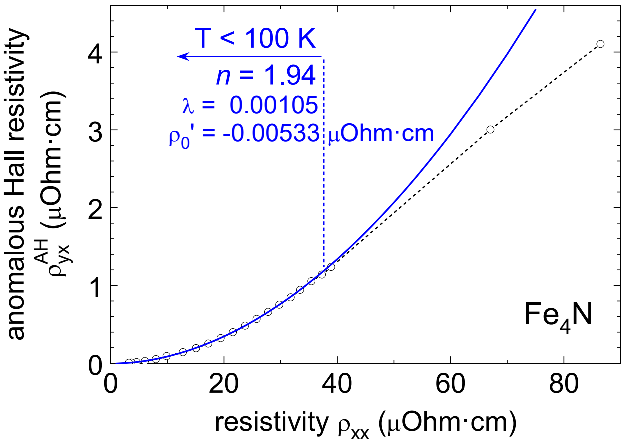

Figure S3. The fit of the relation $\rho_{xy}^{AH} \sim \lambda \rho_{xx}^{n} + \rho_0'$ for Fe_4N.

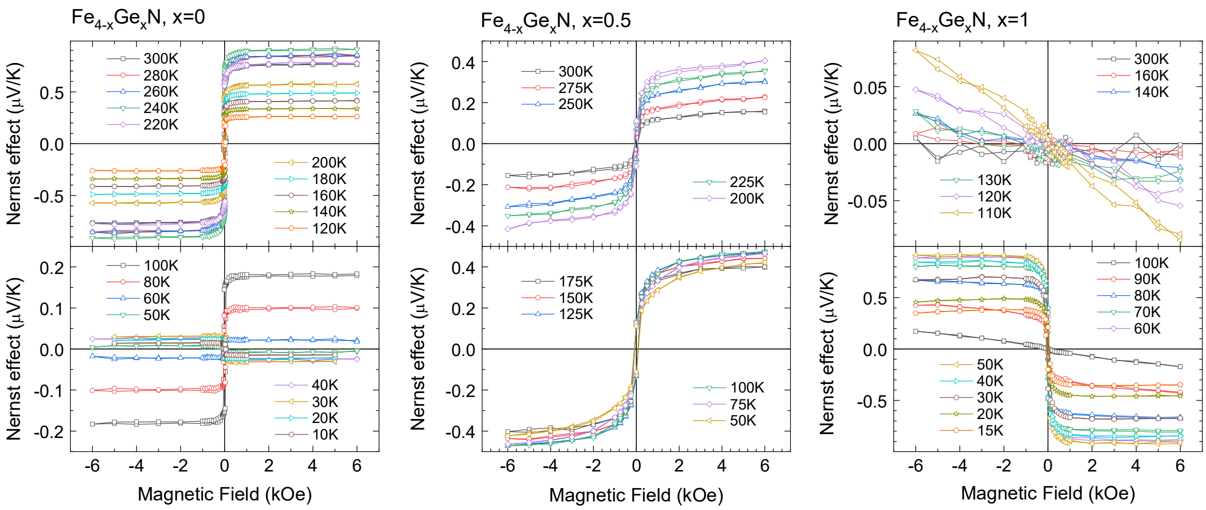

Figure S4. Nernst effect hysteresis loops for $Fe_{4-x}Ge_xN$.

^{57}Fe conversion-electron Mössbauer spectroscopy (CEMS)

In contrast to the traditional transmission Mössbauer spectroscopy, conversion-electron Mössbauer spectroscopy (CEMS) uses the conversion electrons emitted from the sample after the nuclear resonant absorption of the γ photons, with a limited information depth of roughly 200 nm. The CEMS spectra of the $Fe_{4-x}Ge_xN$ samples with x = 0.5 and 1.0 acquired at room temperature are shown in Figure S5, the hyperfine parameters of the fitted components are provided in Table S3. The spectrum of Fe_3GeN at room temperature contains only three doublets D1, D2 and D3, indicating a fully paramagnetic state in accordance with $T_C \approx 100$ K evidenced by temperature-dependent magnetization shown in Figure 6 in the main text. From the comparison of the experimental isomer shift (IS) with DFT calculation results (see Table S4), we can conclude that Ge occupies the *4b* site, related to the large site in the cubic antiperovskite structure of γ'-Fe_4N. Then the two dominating doublets D1 and D2 in the 1:2 ratio correspond to the Fe1c and Fe1ab sites, respectively, forming an elongated octahedron with a nitrogen atom in the center. The higher *IS* than in the γ'-Fe_4N phase, *IS* (Fe1 in Fe_4N) = 0.30 mm/s [17], reflects the decrease in the occupation of (conduction) s-electrons. Fe1c and Fe1ab have remarkably

different electronic properties, *e.g.* they exhibit dominating local and itinerant magnetism in the ground state at low temperatures, respectively [18]. The related variations in electron density are correlated with observed differences in *IS*. Nitrogen vacancies introduce different coordination environments of Fe atoms, resulting in a large number of components in the spectrum with various *IS* and quadrupole splitting (*QS*) values. However, considering the spectrum's shape, these components would exhibit strong correlations when fitted. Finally, a minor doublet D3 can be ascribed to Fe diffused in the Al coating layer.

The spectrum of the $Fe_{3.5}Ge_{0.5}N$ sample still contains a magnetically ordered component, whose magnetic moment (and thereby hyperfine magnetic field at Fe nuclei) fluctuates at the timescale comparable to the characteristic time window of the method, $\tau \sim 10^{-7}$ s. As we can assume a random distribution of Ge among the *4b* site in the $I4/mcm$ structure, obtaining an unambiguous fit remains challenging mainly due to the significant variation in Fe coordination environments, including Ge atoms and potential nitrogen vacancies [19]. Therefore, the magnetic component was approximated by a single sextet (S) within a Blume-Tjon relaxation model, in which the hyperfine magnetic field oscillates between two antiparallel orientations along the EFG principal axis [9]. This component encompasses the signal from Fe with fewer Ge neighbors (both the Fe in *4b* site and their neighboring Fe1) and has a lower isomer shift similar to the undoped γ'-Fe_4N [17]. The fit provided an average frequency of hyperfine-field fluctuations of $1.3(2)\cdot 10^{7}$ s^{-1}. Fe atoms with more Ge neighbors exhibit more rapid magnetic relaxation and manifest as paramagnetic doublets in the spectrum. Given the local similarity of Ge-rich areas to Fe_3GeN, two doublets D1 and D2 in a 1:2 ratio were used to model Fe11- and Fe12-like species, respectively. A higher quadrupole splitting than in Fe_3GeN relates to the reduced symmetry of Fe sites, while a lower average isomer shift is consistent with a higher density of conducting electrons (see Figure S6 for a direct comparison of the spectra). For comparison, we have calculated some of the parameters for Fe_3GeN using DFT method according to procedures applied in [20]. We have considered structural models with Ge in all the Fe1c, Fe1ab and Fe2 sites. A comparison of the experimental and calculated IS clearly confirmed that Ge occupies the Fe2 site.

Table S3. Hyperfine parameters from the fit of room-temperature ^{57}Fe CEMS spectra of the $Fe_{4-x}Ge_xN$ (x = 0.5 and 1) films. *ΔQS* represents the width of the *QS* distribution, B_{hf} the amplitude of the fluctuating hyperfine field, *A* the intensity of the components and Γ_{FWHM} the linewidth. Comparison with DFT calculation.

Sample	Comp.	Site	*IS* (mm/s)	*QS* (mm/s)	*ΔQS* (mm/s)	B_{hf} (T)	Γ_{FWHM} (mm/s)	*A* (%)
Fe_3GeN	D1	Fe1c	0.50(2)	0.40(4)	0.08(3)	-	0.29†	31(1)‡
	D2	Fe1ab	0.41(1)	0.17(2)	0.04(5)	-	0.29†	62(1)‡
	D3	Al(Fe)	0.13(1)	0.39(3)	0.00	-	0.29†	7(1)
$Fe_{3.5}Ge_{0.5}N$	S		0.31(5)	-0.01(5)	-	16.5(8)	0.27*	48(2)
	D1		0.42(2)	0.40(2)	-	-	0.27(2)	17(1)⁑
	D2		0.38(2)	0.52(2)	-	-	0.43(3)	35(1)⁑

*fixed, †, ‡, ⁑ bound parameters

Table S4. Hyperfine parameters of the Fe_3GeN from the DFT calculation. IS represents isomer shift, QS quadrupole splitting, B_{hf} the amplitude of the fluctuating hyperfine field, and A the intensity of the components. Structural parameters were optimized by DFT.

Ge site	Fe Site	*IS* (mm/s)	*QS* (mm/s)	B_{hf} (T)	*A* (%)
Fe2	Fe1c	0.52	0.37	13.4	33
Fe2	Fe1ab	0.40	0.41	4.7	66

Fe1c	Fe2	0.27	0.33	19.1	33
Fe1c	Fe1ab	0.33	0.99	22.9	66
Fe2*	Fe1c*	0.53*	0.15*	8.8*	33*
Fe2*	Fe1ab*	0.45*	0.18*	4.2*	66*
* Structure from Scholz *et al.* [19]					

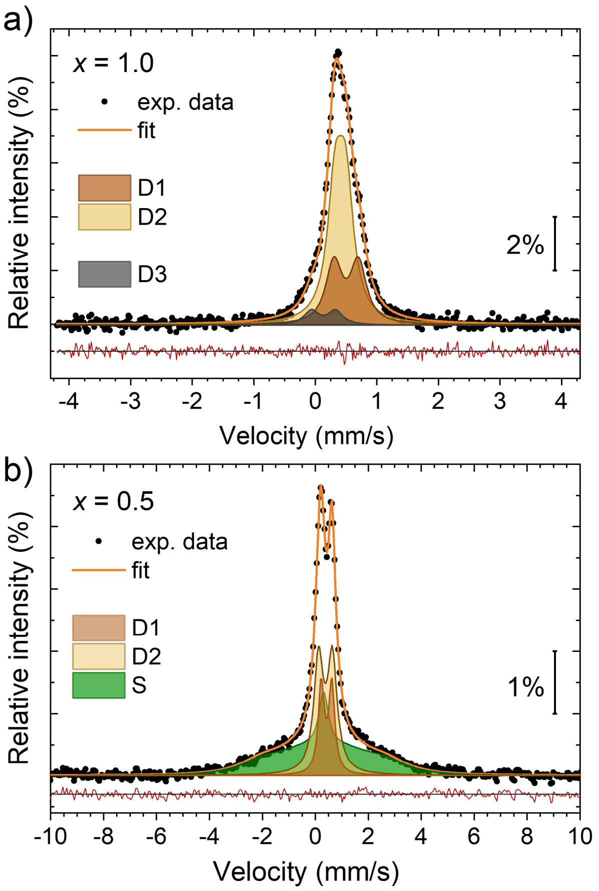

Figure S5. Room-temperature [57]Fe CEMS spectra of the $Fe_{4-x}Ge_xN$ films for (a) x = 1, (b) x = 0.5. The red line shows the difference between the data and the fit (note the different velocity scales).

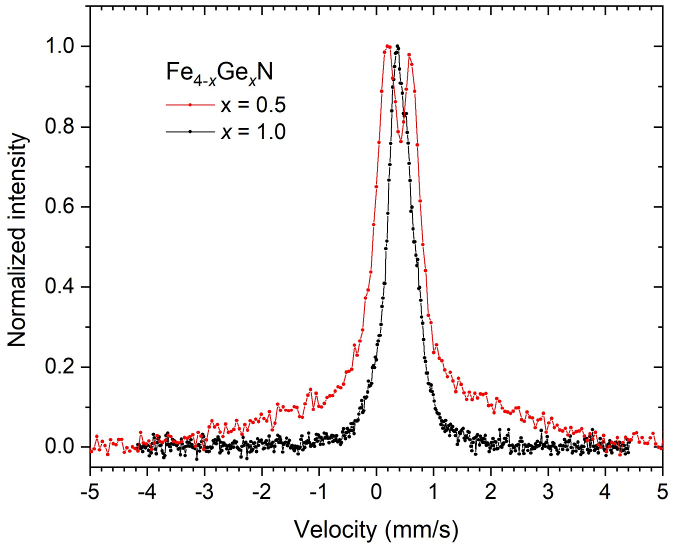

Figure S6. A comparison of room-temperature [57]Fe CEMS spectra of the $Fe_{4-x}Ge_xN$ (x = 0.5, 1.0) films on the MgO substrate, each spectrum was normalized to the maximum intensity.

Modelling magnetism for Fe_3GeN

We quantitatively analyzed 2-component magnetization curves for Fe_3GeN from Figure 10a,b in the main text. Similarly, as for the Hall and Nernst effect (Figure 11a,b in the main text), we decomposed the magnetization curves into 2 components with different saturation fields, and a linear component. For both in-plane (Figure S7a) and out-of-plane (Figure S7b) curves, the fit consistently determined 66% of domains oriented with the *c*-axis perpendicular to the film at several temperatures, while we display a single temperature here. Moreover, the in-plane curves showed that for the rest of 34% domains oriented with *c*-axis in the plane, 17% are in one direction, thus remaining 17% are in the perpendicular direction, showing equal distribution of *c*-axis among the two characteristic directions in the film plane.

Such a quantitative estimate is possible due to an assumption that the saturated signal is the same for both orientations, which is distinct from AHE and ANE, whose magnitudes are generally different, even having opposite signs in our case. The determination of crystallographic domain ratios

from magnetization curves is supposed to be more precise than from XRD, which relies on relative analysis of few diffraction peaks, whose intensities may be affected by different degrees of preference orientation in the epitaxial thin films, while magnetism reflects integral information.

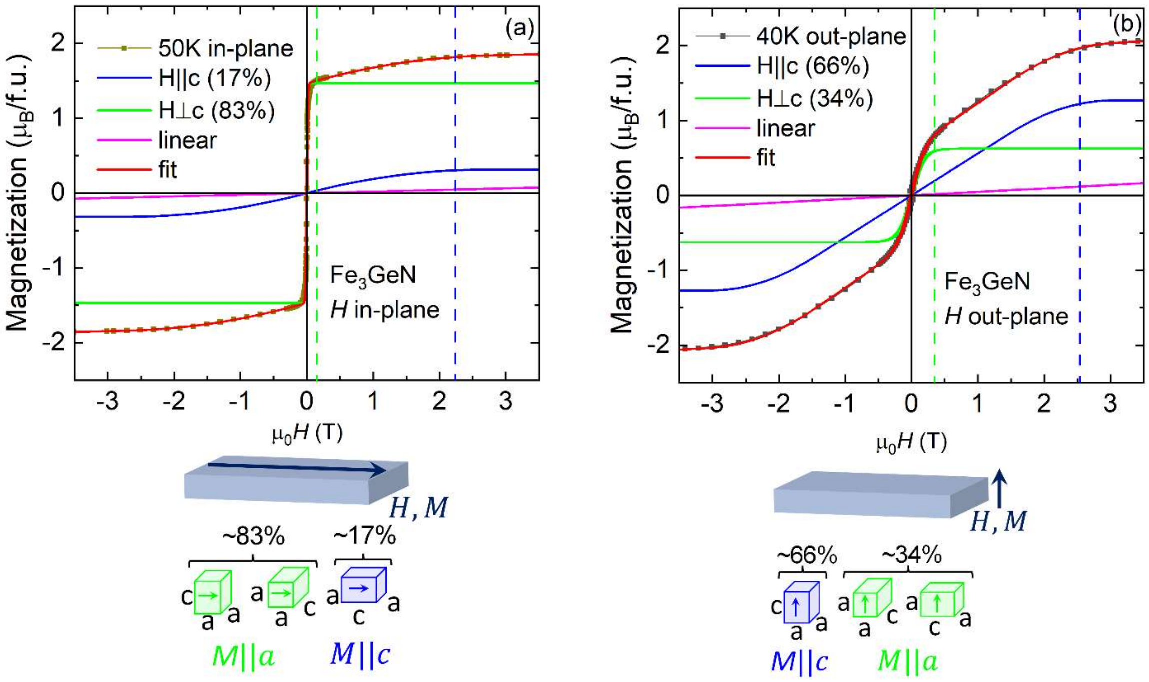

Figure S7. Magnetization curves from Figure 10a,b in the main text at selected temperatures and their decomposition. (a) In-plane and (b) out-of-plane orientation. The blue curves show H||c component with high saturation field, while green components represent H⊥c. The field direction and grain orientations are depicted below with the same color. The linear curves are in magenta and red curves are the sums of all remaining curves, fitting the experimental data.

References

1. M. Klinger. More features, more tools, more CrysTBox, J. Appl. Crystallogr. 50, 1226 (2017). doi:10.1107/S1600576717006793.
2. E.W. Müller, Field desorption, Phys. Rev. 102, 618 (1956). doi:10.1103/PhysRev.102.618
3. D. Blavette, A. Bostel, J.M. Sarrau, B. Deconihout, and A. Menand, An atom probe for three-dimensional tomography, Nature 363, 432 (1993). doi:10.1038/363432a0
4. M. Hans, P. Keuter, A. Saksena, J.A. Sälker, M. Momma, H. Springer, J. Nowak, D. Zander, D. Primetzhofer, and J.M. Schneider, Opportunities of combinatorial thin film materials design for the sustainable development of magnesium-based alloys, Sci. Rep. 11, 17454 (2021). doi:10.1038/s41598-021-97036-6
5. K. Thompson, D. Lawrence, D.J. Larson, J.D. Olson, T.F. Kelly, and B. Gorman, In situ site-specific specimen preparation for atom probe, Ultramicroscopy 107, 131 (2007). doi:10.1016/j.ultramic.2006.06.008
6. M. Hans and J.M. Schneider, Electric field strength-dependent accuracy of TiAlN thin film composition measurements by laser-assisted atom probe tomography, New J. Phys. 22, 033036 (2020). doi:10.1088/1367-2630/ab7770
7. W.C. Oliver and G.M. Pharr, An improved technique for determining hardness and elastic modulus using load and displacement sensing indentation experiments, J. Mater. Res. 7, 1564 (1992). doi:10.1557/JMR.1992.1564
8. T. Žák and Y. Jirásková, CONFIT: Mössbauer spectra fitting program, Surface and Interface Analysis 38, 710 (2006). doi:10.1002/sia2285

9. M. Blume and J.A. Tjon, Mössbauer Spectra in a Fluctuating Environment, Phys. Rev. 165, 446 (1968). doi:10.1103/PhysRev.165.446
10. K. Lagarec and D.G. Rancourt, Recoil - Mössbauer spectral analysis software for Windows, University of Ottawa, Ottawa, ON, Ottawa, 1998. https://denisrancourt.ca/Recoil-Manual.pdf (accessed January 6, 2023).
11. V. Dovale-Farelo, P. Tavadze, L. Lang, A. Bautista-Hernandez, and A.H. Romero, Vickers hardness prediction from machine learning methods. Sci Rep 12, 22475 (2022). doi:10.1038/s41598-022-26729-3
12. A. Šimůnek and J. Vackář, Hardness of Covalent and Ionic Crystals: First-Principle Calculations, Phys. Rev. Lett. 96, 085501 (2006). doi:10.1103/PhysRevLett.96.085501
13. A.I. Yurkova, A.V. Byakova, A.V. Belots'ky, Y.V. Milman, and S.N. Dub, Mechanical Behaviour of Nanostructured Iron Fabricated by Severe Plastic Deformation under Diffusion Flow of Nitrogen, Mater. Sci. Forum 503-504, 645 (2006). doi:10.4028/www.scientific.net/MSF.503-504.645
14. T. Takahashi, J. Burghaus, D. Music, R. Dronskowski, and J. M. Schneider, Elastic properties of γ'-Fe_4N probed by nanoindentation and ab initio calculation, Acta Mater. 60, 2054 (2012). doi:10.1016/j.actamat.2011.12.051
15. S. Zheng, Y. Sun, A. Bloyce, and T. Bell, Characterization of Plasma Nitrided and PVD-TiN Duplex Treated Armco Iron and En40B Steel by Nanoindentation, Mater. Manuf. Process 10, 815 (1995). doi:10.1080/10426919508935068
16. W. Schröter and A. Spengler, Zum E-Modul von nitridhaltigen Schichten auf Stahl, HTM-J. Heat Treat. Mater. 51, 356 (1996). doi:/10.1515/htm-1996-510609
17. J. Vít, P. Levinský, O. Kaman, L. Kubíčková, M. Pashchenko, K. Knížek, M. Jarošová, J. More Chevalier, S. Cichoň, T. Kmječ, J. Kohout, M. Hans, S. Mráz, J.M. Schneider, R.K. Paul, O. Gutfleisch and I. Dirba, Nernst effect in Fe_4N thin layers, *in preparation*.
18. M. Tsumuraya and D.J. Singh, Violation of the rule of parsimony: Mixed local moment and itinerant Fe magnetism in Fe_3GeN, Phys. Rev. B 106, 024408 (2022). doi:10.1103/PhysRevB.106.024408
19. T. Scholz and R. Dronskowski, Structure and magnetism of the solid solution $Ge_xFe_{4-x}N_y$ ($0 \le x \le 1$): from a ferromagnet to a spin glass, J. Mater. Chem. C 5, 166 (2017). doi:10.1039/c6tc04543j
20. R. Řezníček, V. Chlan, H. Štěpánková, P. Novák, J. Żukrowski, A. Kozłowski, Z. Kąkol, Z. Tarnawski, and J. M. Honig, Understanding the Mössbauer spectrum of magnetite below the Verwey transition: Ab initio calculations, simulation, and experiment, Phys. Rev. B 96, 195124 (2017). doi:10.1103/PhysRevB.96.195124
